# Quantitative phase imaging techniques for measuring scattering properties of cells and tissues: a review—part I

**DOI:** 10.1117/1.JBO.29.S2.S22713

**Published:** 2024-07-18

**Authors:** Neha Goswami, Mark A. Anastasio, Gabriel Popescu

**Affiliations:** a University of Illinois Urbana-Champaign, Department of Bioengineering, Urbana, Illinois, United States; b University of Illinois Urbana-Champaign, Department of Electrical and Computer Engineering, Urbana, Illinois, United States

**Keywords:** light scattering, digital holography, quantitative phase imaging

## Abstract

**Significance:**

Quantitative phase imaging (QPI) techniques offer intrinsic information about the sample of interest in a label-free, noninvasive manner and have an enormous potential for wide biomedical applications with negligible perturbations to the natural state of the sample *in vitro*.

**Aim:**

We aim to present an in-depth review of the scattering formulation of light–matter interactions as applied to biological samples such as cells and tissues, discuss the relevant quantitative phase measurement techniques, and present a summary of various reported applications.

**Approach:**

We start with scattering theory and scattering properties of biological samples followed by an exploration of various microscopy configurations for 2D QPI for measurement of structure and dynamics.

**Results:**

We reviewed 157 publications and presented a range of QPI techniques and discussed suitable applications for each. We also presented the theoretical frameworks for phase reconstruction associated with the discussed techniques and highlighted their domains of validity.

**Conclusions:**

We provide detailed theoretical as well as system-level information for a wide range of QPI techniques. Our study can serve as a guideline for new researchers looking for an exhaustive literature review of QPI methods and relevant applications.

## 1 Introduction: Quantitative Phase Imaging

Determination of the 3D refractive index distribution of a thin phase object (transparent to visible light, with negligible absorption) requires precise knowledge of phase information of the scattered field. Traditional brightfield microscopy provides poor contrast for biological samples, such as single cells or thin tissue slices. Zernike phase contrast (PC) microscopy^1^ and differential interference contrast (DIC) microscopy^2^ were the two techniques developed to enhance the contrast for visualization of biological samples. The mode of operation of the above-mentioned techniques is the interference between two field components (scattered and unscattered beam for phase contrast microscopy and two laterally sheared orthogonal polarizations for DIC). Although the contrast is enhanced, the precise reconstruction of the object’s 3D refractive index distribution is still not possible using PC or DIC microscopy alone, as these techniques are qualitative in nature. Accurate phase extraction is made possible today by a wide variety of label-free, quantitative phase imaging (QPI) techniques.^3^^,^^4^ Due to this ability of precise phase retrieval, determination of an object’s 3D refractive index distribution can be achieved. QPI techniques are extremely sensitive to optical pathlength (of the order of nm, both spatially and temporally). These QPI techniques are proving to be highly successful in different realms of biomedicine ranging from characterization of cell membrane fluctuations, transport of cellular materials through organelles, cell growth, etc.^3^

Although extremely sensitive to optical pathlength fluctuations, both spatially and temporally, QPI techniques inherently have lower specificity to the type of subcellular components as compared to fluorescence imaging. To address this issue, researchers have employed deep learning to introduce computational specificity to the phase images.^5^^–^^8^

In this review, we aim to discuss different realizations of 2D QPI techniques as applied to biological specimens. We start with a mathematical discussion of principles of QPI in Sec. 2. We then focus on 2D scattering and present the theoretical framework of 2D scattering from cells and thin tissue slices in Secs. 2.2.1 and 2.2.2. This is followed by a discussion of 2D QPI techniques, such as spatial light interference microscopy (SLIM),^9^ diffraction phase microscopy (DPM),^10^ gradient light interference microscopy (GLIM),^11^ Epi-GLIM,^12^ Hilbert phase microscopy (HPM),^13^ and other digital holographic microscopy (DHM)^14^ techniques in Sec. 2.2.3. Fourier transform light scattering (FTLS)^15^ [an elastic/static light scattering (ELS) technique] is described in Sec. 2.2.4. We conclude with a survey of reported biological applications based on the scattering measurements in Sec. 3.

## 2 Principles of QPI

### 2.1 Mathematical Formulation

Phase retrieval through QPI techniques is possible due to the interference of the reference and object fields. A complex object field (the field that results from the interaction of the incident-illumination field with the object) can be expressed as^3^

$$U(x,y,t)=|U(x,y)|e^{-i[\langle \omega \rangle t-\langle k\rangle \cdot r]},$$ (1)

where the ensemble average of a quantity is denoted by ⟨⟩, the temporal frequency is denoted by ω, and the spatial frequency (wavevector of the field) is denoted by k.

Upon interference of the object field U with a reference $U_{r}=|U_{r}|e^{-i(\langle \omega \rangle t_{r}-k_{r}\cdot r)}$ (assumed to be a plane wave), the resulting irradiance at a detector plane is

$$I_{D}(x,y)=|U(x,y){|}^{2}+|U_{r}{|}^{2}+2|U(x,y)||U_{r}|\cos[\langle \omega \rangle (t-t_{r})-(\langle k\rangle -k_{r})\cdot r+\phi (x,y)],$$ (2)

where ϕ(x,y) denotes the phase difference between the object and reference fields. To retrieve ϕ(x,y), a controlled modification of the total phase is required.

**Fig. 1:**
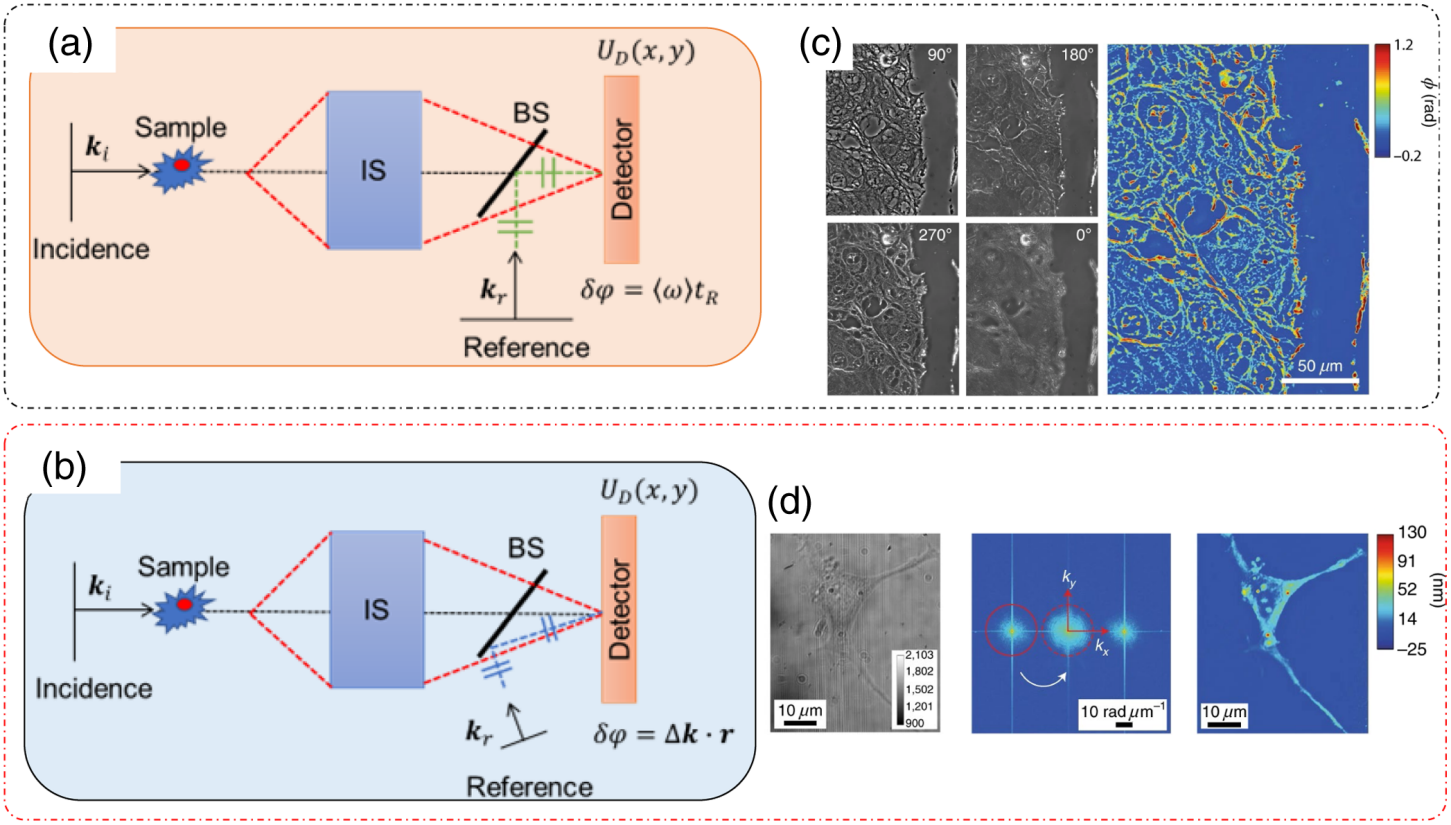
Modes of QPI operation: (a) temporal phase-shifting setup; (b) off axis setup; and (c) phase reconstruction from four temporal phase-shifted measurements in (a), (d). Phase reconstruction from single-shot measurement in (b). IS, imaging system; BS, beam splitter. (a), (b) Adapted from Ref. 16 under CC BY license; (c), (d) reproduced with permission from Ref. 4, © 2018, Springer Nature Limited.

From Eq. (2), we note that there are two controllable quantities that can be modified to introduce desired phase modulations. The first is the temporal modulation of the reference field $t_{r}$. QPI methods that exploit temporal modulations are called (temporal) phase-shifting methods [Figs. 1(a) and 1(c)].^4^^,^^16^ The second is the spatial modulation induced by a tilted reference field, described by wavevector $k_{r}$, which gives rise to another family of QPI techniques called (spatial) off-axis interferometry methods [Figs. 1(b) and 1(d)].^4^^,^^16^

QPI techniques can be compared based on four figures of merit. These are: speed of acquisition (time required to produce one phase image), transverse resolution (maximum spatial frequency coverage), temporal optical pathlength sensitivity (the smallest detectable fluctuations in the optical pathlength at a specific spatial location over time), and spatial optical pathlength sensitivity (the smallest detectable fluctuations in the optical pathlength across a field of view at a constant time).^3^ Temporal phase-shifting QPI methods have superior transverse resolution and spatial optical pathlength sensitivity but have slower rate of acquisition due to the acquisition of multiple intensity frames required to produce one phase image. Hence, for this class of QPI, optimal space-bandwidth product is achieved but with a suboptimal time bandwidth.

Optimal time bandwidth (acquisition rate cut-off determined by the acquisition rate of the camera) can be achieved in off-axis QPI methods because of a single-shot operation. The downside of such off-axis QPI techniques comes in the form of reduction in the space-bandwidth product because of the use of a tilted reference beam. For such systems, the maximum spatial frequency coverage now depends on the angle of tilt in the reference field in addition to the numerical aperture (NA) of the imaging optics.^3^ Thus off-axis QPI techniques provide high time bandwidth but a suboptimal space bandwidth.

Temporal phase noise refers to the temporal fluctuation per pixel in the measured image and is mainly caused by the environmental fluctuations in the optical paths of the two interfering beams, due to vibrations, temperature fluctuation, air current, etc., in addition to the detector temporal noise. To reduce this type of noise in off-axis QPI, a common path geometry is used such that both the object and the reference fields share the same optical path. This geometry results in both fields being degraded by similar noise levels, which can then be suppressed during the phase retrieval process that is described later. This results in improved temporal sensitivity of the QPI system.

Spatial phase noise is defined as the spatial inhomogeneity in the pixel values of the phase image over the entire field of view, which arises due to the speckles generated by imperfections in the optical components along the beam path, such as small scatterers like dust, etc. in addition to the spatial noise introduced by the cameras. To reduce this type of noise, broadband illumination is commonly employed. Such broadband illumination sources have low temporal coherence (few microns), which diminishes the speckles and results in an improved spatial sensitivity.^3^

The signal-to-noise ratio (SNR) of the measured data strongly affects the performance of phase retrieval methods.^17^^,^^18^ For optical imaging, amplitude SNR is defined as |I|/σ, where I is the measured image and σ is its standard deviation representing noise.^3^ The source of noise can be both spatial as well as temporal as discussed above.^19^ To increase the SNR, several techniques can be employed. One method is employing averaging during image acquisition. For incoherent noise (where the noise is uncorrelated between successive frames), the SNR improvement is of the order of $\sqrt{N}$, where N is the number of acquisitions per frame. For coherent noise, due to the correlations between the noise in successive frames, the SNR improvement factor is less than $\sqrt{N}$ and is dependent on the degree of correlation between the noise within repeat acquisitions. Another technique to increase SNR is spatial filtering the images. Caution must be exercised in the choice of the type of spatial filtering (low pass, high pass, or band pass) so as to not to sacrifice the resolution of the image.^3^ SNR can also be increased using a high-power illumination source, but care must be exercised in adjusting the exposure time as photodamage to the sample can occur. A better way to increase SNR is noise reduction, using noise-efficient or low-noise detectors^20^ or utilizing computational denoising techniques.^21^^–^^25^ An interesting development to offset temporal noise using the intensity correlations as the measured parameter has been recently reported in literature as a phase imaging method amenable to low-light conditions.^26^

### 2.2 2D Scattering, QPI Geometries, Fourier Transform Light Scattering

In this section, we discuss some QPI techniques for extracting 2D light scattering signals and describe their applications.

#### 2.2.1 Scattering from thin tissue slices under the Born approximation

The linear interaction of light with matter results in two optical phenomena: absorption and scattering. The relative strength of these interactions is determined by the optical properties of the object, i.e., the imaginary and real parts of the refractive index as well as the object’s thickness. Solving inverse problems to determine the underlying refractive index distribution gives insight into the object structure. Here we focus on optical methodologies that can recover the scattering properties via quantitative phase measurements and estimate structural information of the object using tomographic techniques. However, before discussing these, a description of the physical meaning of the phase measurements in forward and backward geometries is provided.^27^

**Fig. 2:**
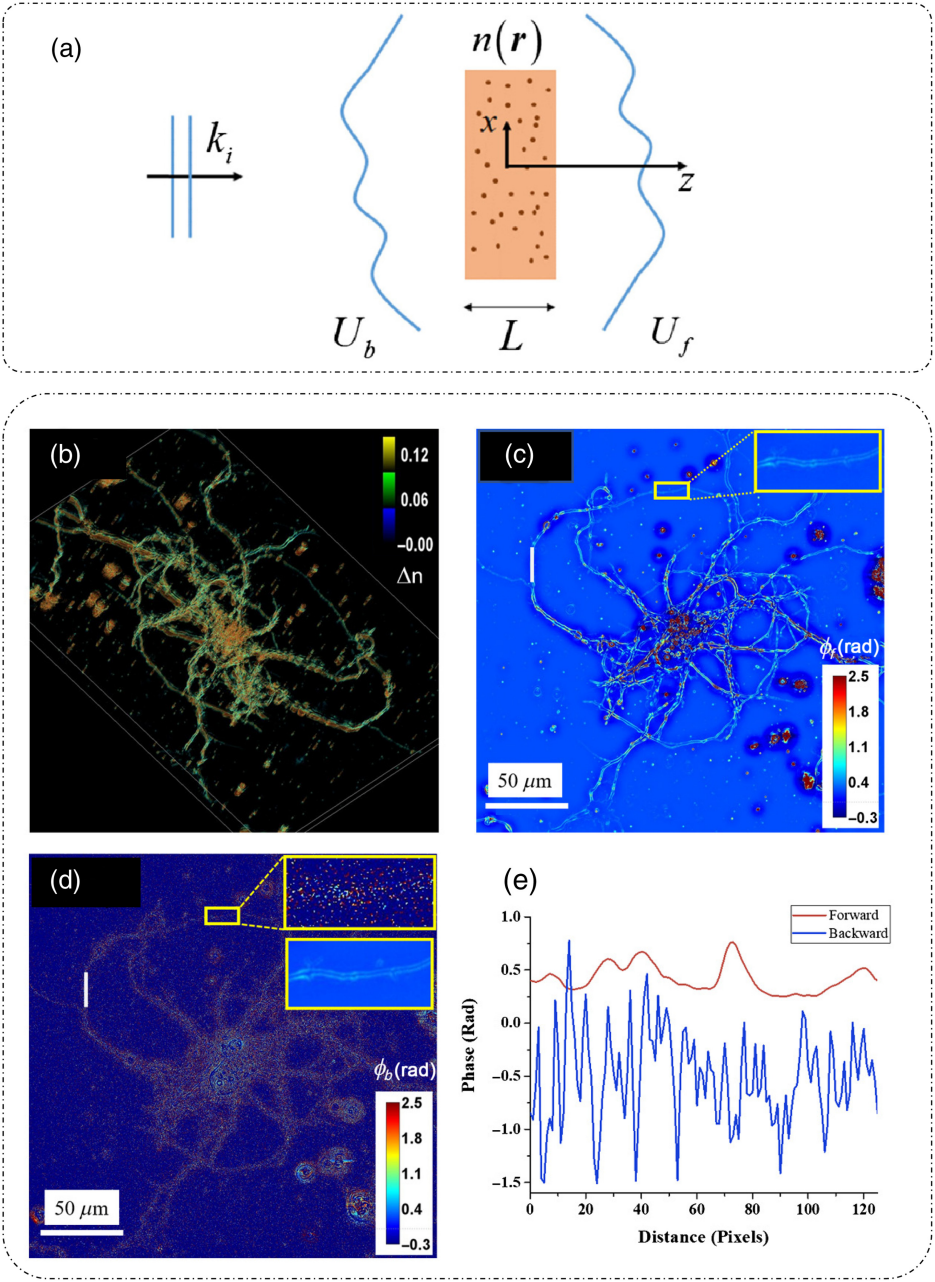
Forward and backscattering phase: (a) scattering geometry; (b) refractive index distribution of a neuron from forward scattering measurements; (c) calculated phase measurements in forward scattering mode; (d) calculated phase map for backscattering mode; and (e) phase profiles along the white lines in (c) and (d). (a)–(e) Reproduced with permission from Ref. 27, © 2017, Optical Society of America (Optica).

QPI techniques provide highly sensitive optical phase measurements in both transmission and reflection geometries.^3^ When the incident light associated with a wavevector $k_{i}$ interacts with a scattering sample characterized by an inhomogeneous refractive index distribution n(r) and thickness L, it is scattered in both forward and backward directions [Fig. 2(a)].^27^ The meaning of the wavefield phase in forward scattering measurements is straightforward. Under weakly scattering conditions that are satisfied by thin biological samples, it is the pathlength accumulation encountered by the incident light upon propagation through the object that gives rise to the phase measurements. As such, the measurement in transmission as shown in Figs. 2(b) and 2(c)^27^ is related to the optical pathlength or the product of refractive index difference and depth of the object. Thus from forward scattering measurements, we can recover the structural information of the object with high accuracy. Differently, the backscattered phase [Fig. 2(d)] originates from the interference of the backscattered waves from different depths within the object.^27^

To mathematically describe the forward and backscattered field components, consider the geometry in Fig. 2(a).^27^ A plane wave with wavevector $k_{i}=\beta_{0}\hat{z}$ is incident on an object with refractive index n(r) and thickness L. The quantities $U_{b}$ and $U_{f}$ denote the backscattered and forward scattered scalar fields, respectively. Consider the inhomogeneous Helmholtz equation:

$$\nabla^{2}U_{1}(r,\omega )+n_{0}^{2}\beta_{0}^{2}U_{1}(r,\omega )=-\beta_{0}^{2}\chi (r,\omega )U_{0}(r,\omega ),$$ (3)

where $U_{0}(r,\omega )=A(\omega )e^{in_{0}\beta_{0}z}$ is the incident plane wave along the z axis, $U_{1}$ is the scattered field, $n_{0}$ is the refractive index of the surrounding medium, $\chi (r,\omega )=n^{2}(r,\omega )-n_{0}^{2}$ is the scattering potential of the object, and $\beta_{0}=\omega /c$ is the wavenumber in vacuum. Following the Fourier transform procedure outlined in Ref. 27, the forward scattered measurement $U_{f}$ can be expressed as the sum of the incident and scattered light as

$$U_{f}(k_{⊥},z,\omega )=A(\omega )\delta (k_{⊥})e^{i\beta z}-i\beta_{0}^{2}A(\omega )\frac{e^{i\gamma z}}{2\gamma }\chi (k_{⊥},\gamma -\beta ,\omega ),$$ (4)

where k⊥=kxx^+kyy^, $\gamma =\sqrt{\beta^{2}-k_{⊥}^{2}}$, and $\beta =n_{0}\beta_{0}$, $k_{x}$ and $k_{y}$ denote the wavevectors in the transverse (x and y) directions, respectively, and δ denotes the Dirac delta function. Similarly, the detected field in the backscattered geometry can be expressed as

$$U_{b}(k_{⊥},z,\omega )=A(\omega )\delta (k_{⊥})e^{-i\beta z}+i\beta_{0}^{2}A(\omega )\frac{e^{-i\gamma z}}{2\gamma }\chi (k_{⊥},-\gamma -\beta ,\omega )-A(\omega )\delta (k_{⊥})e^{-i\beta z}.$$ (5)

Note that there is no incident field in backscattered measurement. Also note that Eq. (5) includes an addition and subtraction of the incident field (first and third terms). The second term on the right hand side of Eq. (5) is the backscattered solution of Eq. (3).

Under the small angle approximation, γ≈β, transforming Eqs. (4) and (5) to the spatial domain leads to the following expressions:

$$U_{f}(r_{⊥},z,\omega )=A(\omega )e^{i\beta z}-\frac{i}{2n_{0}}\beta_{0}A(\omega )e^{i\beta z}\int_{-L/2}^{L/2}{\left[n^{2}(r_{⊥},z,\omega )-n_{0}^{2}]e^{-ik_{z}z}dz\right|}_{k_{z}=0},$$ (6)

$$U_{b}(r_{⊥},z,\omega )=A(\omega )e^{-i\beta z}+\frac{i}{2n_{0}}\beta_{0}A(\omega )e^{-i\beta z}\int_{-L/2}^{L/2}{\left[n^{2}(r_{⊥},z,\omega )-n_{0}^{2}]e^{-ik_{z}z}dz\right|}_{k_{z}=-2\beta }-A(\omega )e^{-i\beta z}.$$ (7)

Since for most biological samples, the refractive index contrast between the surrounding media and object is low, $n^{2}(r,\omega )-n_{0}^{2}$ can be approximated as $2n_{0}(n(r,\omega )-n_{0})$ and Eqs. (6) and (7) can be simplified to^27^

$$U_{f}(r_{⊥},z,\omega )=A(\omega )e^{i\beta z}e^{-i\beta_{0}[\overset{¯}{n}(r_{⊥},\omega )-n_{0}]L},$$ (8)

$$U_{b}(r_{⊥},z,\omega )=A(\omega )e^{-i\beta z}e^{i\beta_{0}\int_{-L/2}^{L/2}[n(r_{⊥},z,\omega )-n_{0}]e^{i2\beta z}dz}-A(\omega )e^{-i\beta z}.$$ (9)

Here $\overset{¯}{n}(r_{⊥},\omega )=\frac{1}{L}\int_{-L/2}^{L/2}n(r_{⊥},z,\omega )dz$ is the refractive index averaged along the z direction. These equations show that, in the transmission geometry, the argument of the second exponential in Eq. (8) gives the phase shift induced by the object $\phi =\beta_{0}[\overset{¯}{n}(r_{⊥},\omega )-n_{0}]L$. However, from Eq. (9), there is no such straightforward explanation of the phase in the backscattered field. From the argument of the second exponential in the first term of Eq. (9), the phase difference is the weighed axial projection of refractive index shift with the weighing factor being $e^{i2\beta z}$, which is the accumulated phase at different depths. Thus the phase in backscattering arises from the superposition of plane waves, backscattered from different depths within the object.^27^

The 3D refractive index distribution of a sample imaged through SLIM is shown in Fig. 2(b).^27^ Figures 2(c) and 2(d) show the phase measurements reported in Ref. 27 for forward scattering and backscattering. SLIM images were used to obtain the forward scattered phase map and using the mathematical relations outlined in Ref. 27 and mentioned in detail in “Spatial light interference microscopy” section, the backscattered phase map was obtained. As evident in Fig. 2(e), there is a considerable difference between forward scattered and backscattered phase measurements.^27^

#### 2.2.2 Scattering phase theorem

The scattering parameters of an object include the scattering mean free path $l_{s}$ and the scattering anisotropy factor g. The scattering mean free path $l_{s}$ represents the mean length between two scattering incidents and the anisotropy factor g describes the directivity of the scattering or the average cosine of the scattering angle. Traditionally, the estimation of these parameters requires experiments combined with diffusion models or Monte Carlo simulations.^28^^–^^30^

The scattering phase theorem^31^ connects the phase measurements for thin tissue slices to the scattering parameters of the bulk object through simple mathematical formulas. Mathematical relations between the phase ϕ, scattering mean free path $l_{s}$, and scattering anisotropy factor g were derived in Ref. 31. The reported results allow one to extract the corresponding $l_{s}$ and g maps from experimental phase measurements.^31^

**Fig. 3:**
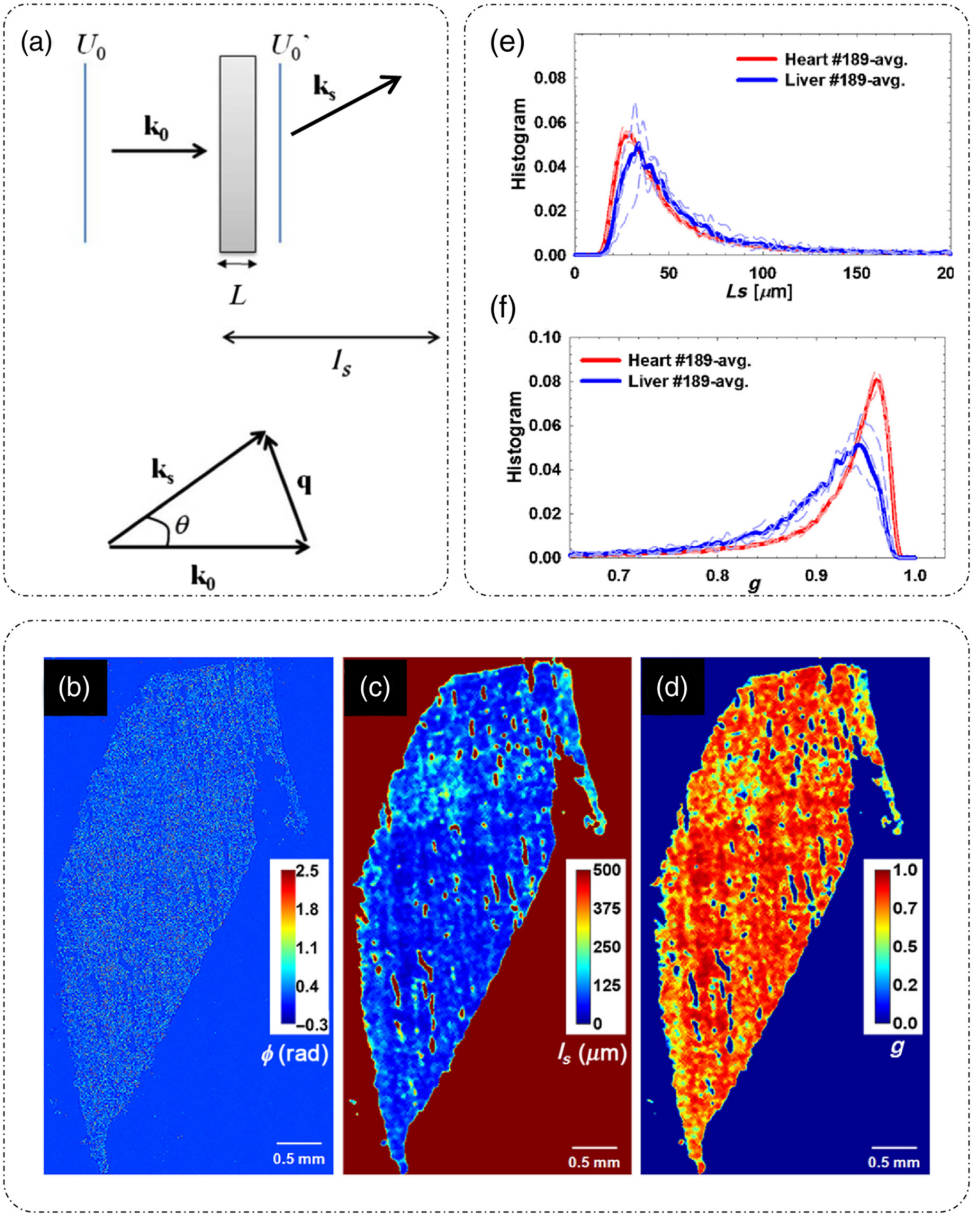
Scattering from thin tissue slices: (a) scattering geometry for a sample of thickness L; (b) phase map of entire rat liver tissue slice; (c) extracted scattering mean free path map; (d) extracted scattering anisotropy map; (e) histograms of scattering mean free path for heart versus liver; and (f) histograms of scattering anisotropy for heart versus liver. (a) Adapted with permission from Ref. 31, © 2011, Optica; (b)–(f) reproduced with permission from Ref. 32, © 2011, Optica.

To derive the scattering phase theorem relations, it was assumed (in Ref. 31) that there is no effect of absorption; the sample is thus a phase object and hence satisfies the first-order Born approximation. It was also assumed that the phase shift is random and follows Gaussian distribution. By the use of the geometry shown in Fig. 3(a)^31^ and following the discussion in the previous section, the measured field in the transmission geometry, $U_{f}$, is the sum of the incident (unscattered) field $U_{0}^{\prime }$ and forward scattered field $U_{1}^{\prime }$:

$$U_{f}(r)=U_{0}^{\prime }+U_{1}^{\prime }(r)=U_{0}e^{i\phi (r)},$$ (10)

where ϕ is the phase information extracted from the intensity measurements. The incident field $U_{0}^{\prime }$ represents a spatially homogenous or an average (DC) term (here and elsewhere in this paper, we use DC to indicate quantity at zero-spatial frequency or an average term), which represents ballistic or unscattered component of total detected field. The DC term $U_{0}^{\prime }$ can hence be calculated as the spatial average of detected field (spatial averaging removes the dependence on spatial coordinate and hence makes the term a constant in space), $U_{f}$, so $U_{0}^{\prime }=\langle U_{0}e^{i\phi (r)}\rangle_{r}$.

Considering that the probability density function of phase shifts follows a Gaussian distribution, the DC term $U_{0}^{\prime }$ can be calculated as

$$U_{0}^{\prime }=\frac{U_{0}}{\sqrt{2\pi \langle \Delta \phi^{2}\rangle_{r}}}\int_{-\infty }^{\infty }e^{i\phi }e^{-\frac{\phi^{2}}{2\langle \Delta \phi^{2}\rangle_{r}}}d\phi =U_{0}e^{-\frac{\langle \Delta \phi^{2}\rangle_{r}}{2}},$$ (11)

where $\langle \Delta \phi^{2}\rangle_{r}$ denotes the variance of phase shift. Taking square of both sides in Eq. (11) to get the irradiance relationship $I_{0}^{\prime }=I_{0}e^{-\langle \Delta \phi^{2}\rangle_{r}}$ and comparing with Lambert–Beer law $I_{0}^{\prime }=I_{0}e^{-L/l_{s}}$, the scattering mean free path $l_{s}$ can be expressed as^31^

$$l_{s}=\frac{L}{\langle \Delta \phi^{2}(r)\rangle_{r}}.$$ (12)

This equation relates the scattering mean free path $l_{s}$ to the variance in the measured phase shift $\langle \Delta \phi^{2}(r)\rangle_{r}$ through an inverse relationship. It implies that for objects with large variance in phase have shorter $l_{s}$. This draws an intuitive picture, in the sense that high variance in phase suggests large inhomogeneity in the sample that causes higher scattering and low scattering mean free path.

The scattering anisotropy factor g denotes the directionality of the scattering events and is defined as the angular mean of cosine of the scattering angle $g=\langle \cos\;\theta \rangle_{\theta }$. The scattering angle θ can be expressed in terms of the vector difference between incident wavevector $k_{0}$, with $|k_{0}|=\beta_{0}$ and the scattered wavevector $k_{s}$, with $|k_{s}|=\beta_{0}$, the difference termed as momentum transfer q:

$${q=k}_{s}-k_{0},\;q=\sqrt{q_{x}^{2}+q_{y}^{2}},$$ (13)

$$q=2\beta_{0}\;\sin(\frac{\theta }{2}),\;\;\cos\;\theta =1-\frac{q^{2}}{2\beta_{0}^{2}}.$$ (14)

The probability distribution function for the angular scattering can be expressed as^31^

$$P(q_{x},q_{y})=\frac{|\overset{˜}{U_{f}}(q_{x},q_{y}){|}^{2}}{\iint |\overset{˜}{U_{f}}(q_{x},q_{y}){|}^{2}dq_{x}\;dq_{y}}.$$ (15)

Since the mean of a random function f(x) with probability density function g(x) can be calculated as $\langle f(x)\rangle_{x}=\int f(x)g(x)dx$, using Eqs. (14) and (15), g can be expressed as

$$g=\frac{\int_{-1}^{1}\cos(\theta )p[\cos(\theta )]d[\cos(\theta )]}{\int_{-1}^{1}p[\cos(\theta )]d[\cos(\theta )]}=1-\frac{\langle |\nabla [\phi_{ls}(r)]{|}^{2}\rangle_{r}}{2\beta_{0}^{2}},$$ (16)

where $\langle |\nabla [\phi_{ls}(r)]{|}^{2}\rangle_{r}$ is the variance of the gradient of measured phase for an object of thickness $l_{s}$. For an object thickness L, which is much smaller than the scattering mean-free path $l_{s}$, i.e., for weakly scattering samples, g can be written as^31^

$$g=1-{\left(\frac{l_{s}}{L}\right)}^{2}\frac{\langle |\nabla [\phi (r)]{|}^{2}\rangle_{r}}{2\beta_{0}^{2}}.$$ (17)

Using Eq. (12), g can be expressed in terms of phase alone as^31^

$$g=1-\frac{1}{2\beta_{0}^{2}}\frac{\langle |\nabla [\phi_{ls}(r)]{|}^{2}\rangle_{r}}{\langle \Delta \phi^{2}(r)\rangle_{r}^{2}}.$$ (18)

This expression states that the higher the ratio of variance of phase gradient to the phase variance, the smaller the g is and the higher the scattering angle θ is.^31^

These relationships in Eqs. (12) and (18) that relate the measured phase and scattering parameters are referred to as the scattering-phase theorem.^31^ The significance of this theorem lies in the fact that to determine the scattering parameters of an object, measurement of phase perturbations introduced by the object is sufficient. The effects of frequency averaging of the measured scattering parameters due to broadband illumination and limited NA of the objective are discussed in Ref. 32.

Experimental corroboration of the scattering phase theorem is provided in Ref. 32, where SLIM, a form of QPI discussed in detail later, is used to quantitatively measure the phase of entire rat liver tissue slice of depth 5  μm. The phase map is shown in Fig. 3(b).^32^ Using Eqs. (12) and (18), maps of scattering mean free path $l_{s}$ and scattering anisotropy g are shown in Figs. 3(c) and 3(d), respectively.^32^ Histograms of the scattering parameter measurements for heart and liver tissue slices are shown in Figs. 3(e) and 3(f),^32^ which show that the heart tissue is a stronger scatterer as compared to the liver tissue and that the scattering is mostly forward in heart tissue, as g is nearing unity. These measurements are consistent with the traditional diffusion scattering measurements.^32^

#### 2.2.3 Transmission/reflection geometries

##### Spatial light interference microscopy

**Fig. 4:**
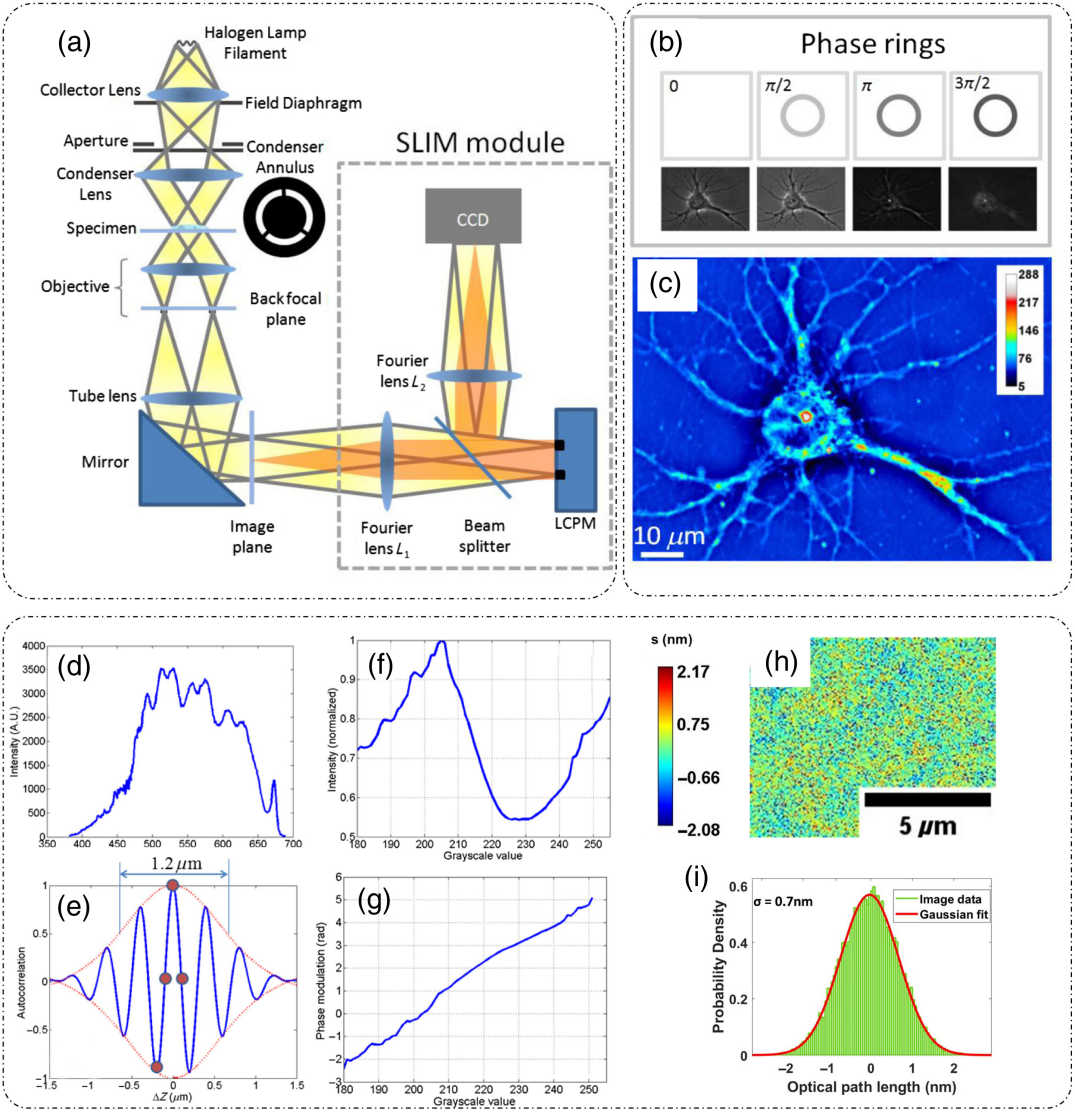
SLIM principle and operation: (a) SLIM system setup; (b) four-phase shifted intensity measurements; (c) resulting SLIM optical pathlength map; (d) spectrum of broadband illumination source; (e) autocorrelation curve for the spectrum shown in (d); (f) calibration curve showing the captured intensity versus grayscale value fed to the LCPM; (g) phase calibration curve for the LCPM extracted from (f); (h) optical pathlength fluctuation map for a sample-less field of view; and (i) histogram of optical pathlength fluctuations in (h) showing the spatial optical pathlength sensitivity of SLIM to be 0.7 nm. (a)–(g) Reproduced with permission from Ref. 9, © 2011, Optica; (h), (i) reproduced from Ref. 33 under CC BY license.

SLIM is a temporal phase-shifting QPI technique that enables the extraction of the phase delay introduced by the sample.^9^ SLIM is based on the principles of phase contrast microscopy (for contrast enhancement of weak scattering biological samples) and Gabor’s holography (for phase extraction) and is implemented in a common path phase-shifting geometry. The optical setup for SLIM is shown in Fig. 4(a).^9^ A SLIM module is installed at the output port of a standard phase contrast microscope, which includes broadband annular illumination and a phase contrast objective lens. The Fourier lens $L_{1}$ performs the spatial Fourier transform on the output from the phase contrast microscope. This spatial frequency content is projected on a liquid crystal phase modulator (LCPM) or a reflective, electrically addressed spatial light modulator (SLM), which provides external phase modulations between the reference and object fields. The Fourier lens $L_{2}$ projects the frequency content back to spatial domain and an intensity image is captured at the camera (CCD or sCMOS) [Fig. 4(a)].^9^

As described earlier, the complex field on the image plane (where the image forms) after passing through a weakly scattering sample can be expressed as a sum of an incident reference field $U_{0}^{\prime }$ and a scattered object field $U_{1}^{\prime }(x,y)$.^9^^,^^34^

The recorded intensity at the detector (camera) plane (for collinear reference and object fields) can be expressed as

$$I(x,y;\varphi )=|U_{0}^{\prime }{|}^{2}+|U_{1}^{\prime }(x,y){|}^{2}+2|U_{0}^{\prime }||U_{1}^{\prime }(x,y)|\cos(\Delta \phi (x,y)+\varphi ).$$ (19)

Here Δϕ(x,y) is the phase difference between the reference and the object fields, and φ is the external phase shift (applied to the reference field) that is modulated in steps of π/2 to enable phase extraction [Fig. 4(b)].^9^ These phase modulations are temporal in nature and represent the first term of the cosine ($\left\langle \omega \rangle (t-t_{r}\right)$) in Eq. (2). For each of the four temporal phase modulation steps corresponding to φ=0,π/2,3π/2, and π, individual intensity frames are recorded. The phase difference Δϕ(x,y) is extracted using the phase-shifting algorithm^35^ and is expressed as^9^^,^^34^

$$\Delta \phi (x,y)=\tan^{-1}[\frac{I(x,y;-\pi /2)-I(x,y;\pi /2)}{I(x,y;0)-I(x,y;\pi )}].$$ (20)

Considering the field amplitude division factor $\xi =|U_{1}^{\prime }(x,y)|/|U_{0}^{\prime }|$, the phase of the complex field is expressed as^9^^,^^34^

$$\phi (x,y)=\tan^{-1}[\frac{\xi (x,y)\sin(\Delta \phi (x,y))}{1+\xi (x,y)\cos(\Delta \phi (x,y))}].$$ (21)

Optical pathlength maps can also be extracted from the measured phase shifts (substituting the wavelength with the central wavelength for broadband illumination) as shown in Fig. 4(c).^9^ A uniform background of the phase/optical pathlength maps due to the suppression of speckles is achieved due to the broadband illumination used in SLIM. The spectrum and the derived temporal autocorrelation for such a source is shown in Figs. 4(d) and 4(e), respectively.^9^

During calibration, the dependence of the phase shift introduced by the SLM on the input gray level values is determined. The SLM is operated in the amplitude modulation mode by placing it in between two crossed polarizers. The SLM is fed discrete gray level values in the range of 0 to 511 and the corresponding intensity image is recorded [Fig. 4(f)].^9^ The sine component of the complex exponential signal is calculated using Hilbert transform of the recorded intensity, which is an interferogram with a DC and a cosine component, and the phase is extracted [Fig. 4(g)].^9^

**Fig. 5:**
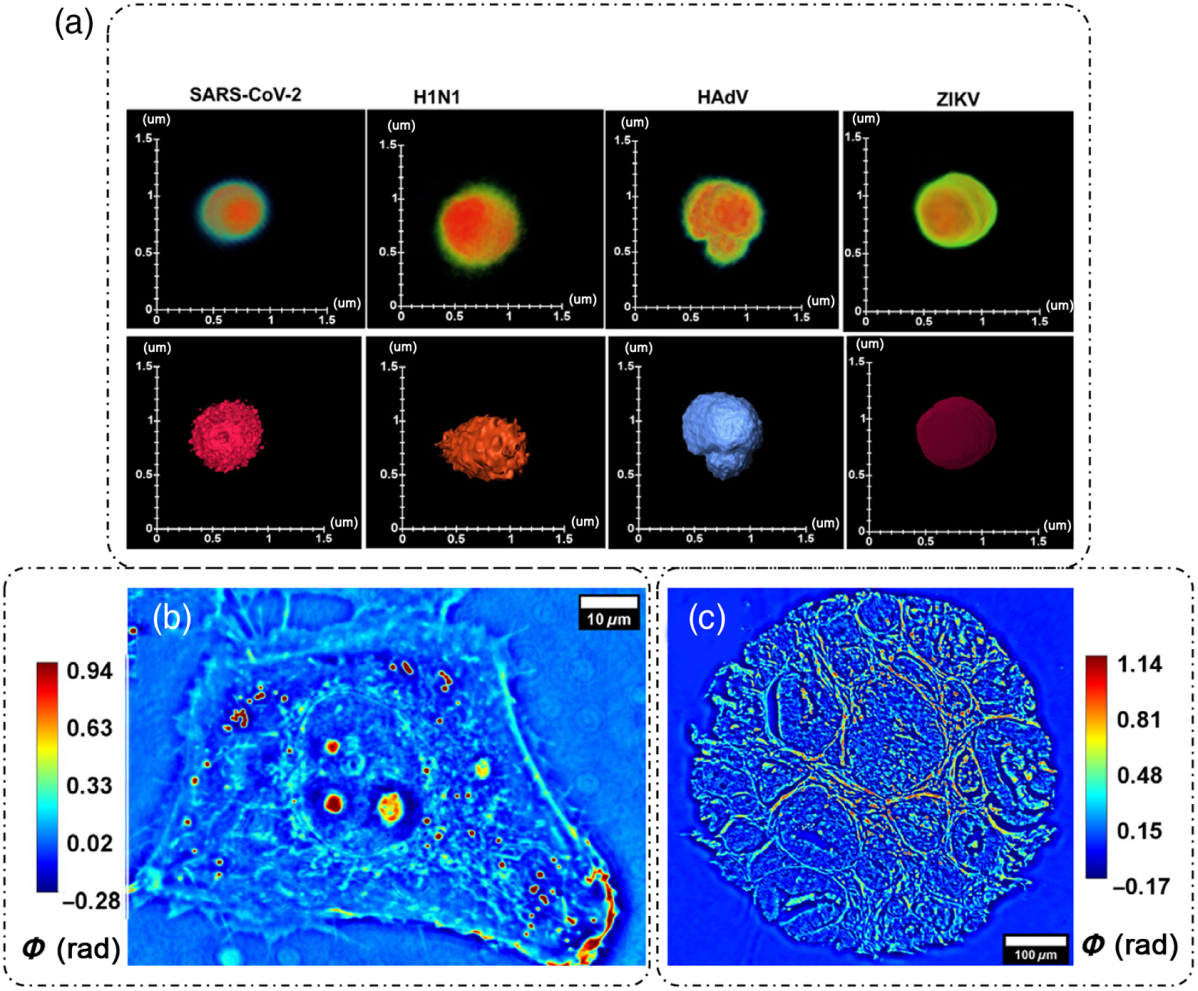
SLIM applications: (a) virology: the top row is the volumetric and the bottom row is the surface reconstruction from the SLIM images of SARS-CoV-2, H1N1, HAdV, and ZIKV particles, respectively. (b) Single cell imaging and intracellular organelle detection and (c) whole tissue imaging. (a) Reproduced from Ref. 33 under CC BY license.

Figures 4(h) and 4(i) show the spatial optical pathlength sensitivity of the SLIM system.^33^ Temporal sensitivity (defined as the standard deviation of the optical pathlength fluctuations at a specific spatial location over time) was characterized by a time-lapse measurement of the same $10\times 10\;\mu m^{2}$ field of view for 256 frames and was found to be 0.03 nm.^9^ The spatial sensitivity (defined as the standard deviation of the optical pathlength fluctuations over space) was characterized to be <1  nm.^9^^,^^33^ SLIM’s high optical pathlength sensitivity makes it an ideal choice of instrument for multiscale applications, such as diffraction-limited virus imaging [Fig. 5(a)],^33^ single cell and intracellular organelle imaging [Fig. 5(b)], blood smears for pathology studies,^36^ and tissue imaging [Fig. 5(c)].

Fourier phase microscopy (FPM) is the predecessor of SLIM.^37^ Its principle of operation is similar to SLIM except for the use of an annular illumination and a base phase contrast microscope in the case of SLIM. FPM and its later variants were developed as an add-on module for brightfield microscopes and have been employed successfully for evaluation and monitoring of cell growth and cellular dynamics.^38^

##### Diffraction phase microscopy

**Fig. 6:**
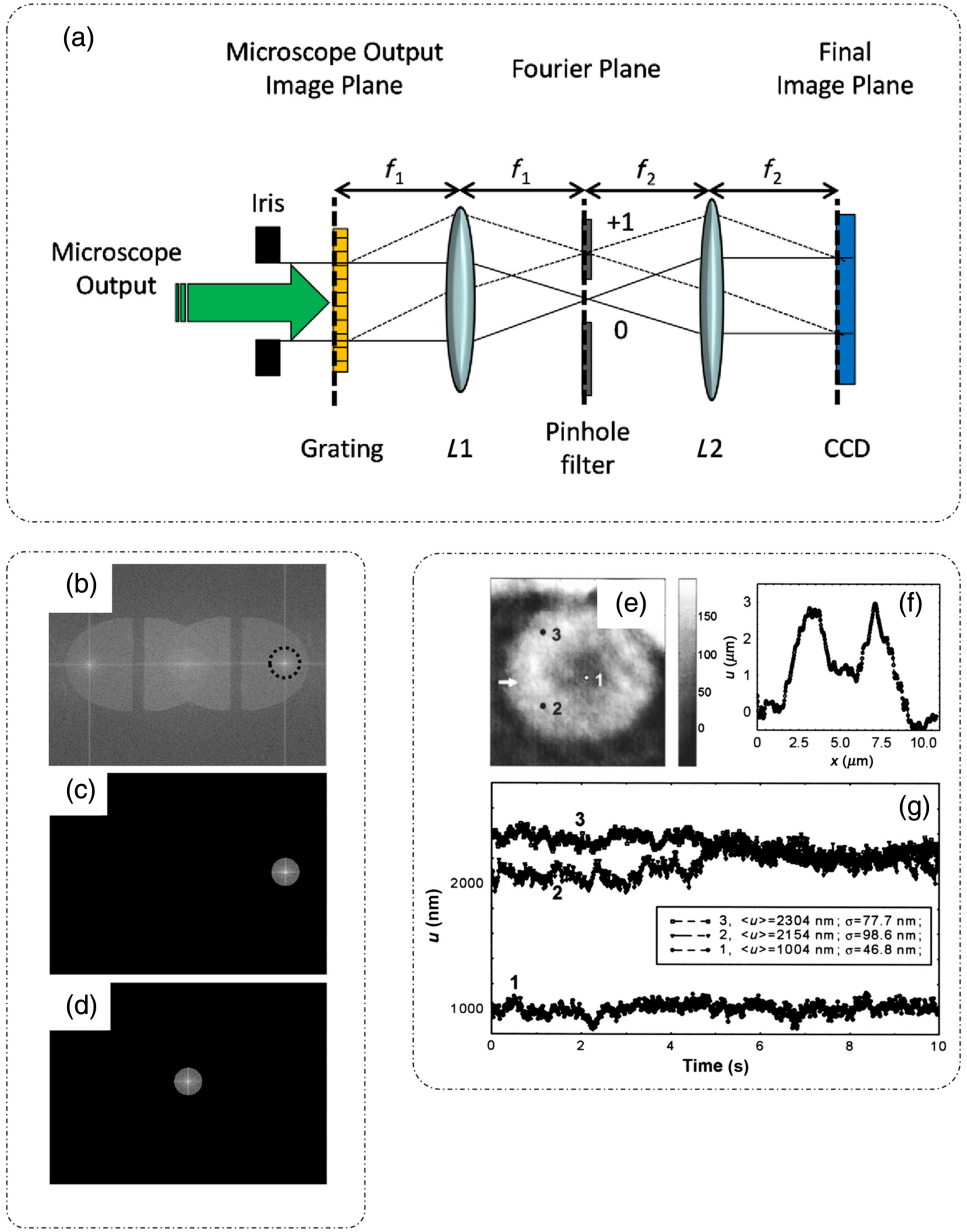
Diffraction phase microscopy: principle of operation, reconstruction, and application: (a) optical setup for DPM; (b) spatial frequency information of the interferogram; (c) single side-band is selected after bandpass filtering (b); (d) band-passed frequency domain image, (c) is translated to DC; (e) optical pathlength map of RBC obtained with DPM; (f) thickness profile through the center of the cell indicated by arrow in (e); and (g) nanometer scaled temporal fluctuations in the membrane thickness observed at three points marked in (e) through DPM. (a)–(d) Reproduced with permission from Ref. 43, © 2014, Optica; (e)–(g) reproduced with permission from Ref. 10, © 2006, Optica.

DPM is an off-axis technique, and thus requires only a single shot measurement to extract the phase map of the sample.^10^^,^^39^^–^^43^ It is a common path instrument based on Mach–Zehnder interferometric configuration, which employs spatial modulation to extract phase information. The DPM optical setup is shown in Fig. 6(a), as a module installed at the output port of a standard optical microscope.^43^ At the image plane, a grating is placed that separates the light into different diffraction orders. Each diffraction order carries full image information.^10^^,^^43^

The field at the diffraction grating plane can be written as^43^

$$U_{G}(x,y)=U_{0}(x,y)+U_{1}(x,y)e^{i\alpha x},$$ (22)

where $U_{0}(x,y)$ is the zeroth order and $U_{1}(x,y)$ is the first-order diffracted field. At the focal plane of lens $L_{1}$, a custom spatial filter is placed that passes the zeroth-order diffraction beam and filters the first-order diffraction beam, whereas blocking all other orders of diffraction. Lens $L_{2}$ performs another Fourier transform to make the first order approximately a plane wave at CCD plane. This homogenous first-order diffraction beam serves as the reference beam and interferes with the zeroth order object beam that contains information of the sample. The interferogram is recorded, 2D Fourier transformed, and spatially filtered around $k_{x}=\alpha$, with a radius of $k_{\max}=\beta_{0}{\mathrm{NA}}_{\mathrm{obj}}$, to extract the cosine term. The extracted frequency content is translated back to the frequency origin (DC), as can be seen in Figs. 6(b)–6(d).^43^ The spatial filter is apodized with a Gaussian kernel to avoid ringing in the final image. This procedure is repeated for both sample image and a sample-less background calibration image. After the inverse FFT, the two complex fields are divided to remove the background phase, and the phase information is recovered by taking the argument of the complex field.^43^ In addition to the above-described method for phase extraction from off-axis measurements, several other computational techniques are used in the literature like integral transforms, derivative methods, etc.^43^^–^^45^ Thus the phase extraction is a single shot, and the throughput is only limited by the acquisition rate of the CCD.^10^ The spatial and temporal optical pathlength sensitivities of DPM is 3 and 0.6 nm, respectively.^43^

The high optical pathlength sensitivity and single shot measurements enable DPM to be used for the measurement of fast dynamic processes, such as measurements of membrane fluctuations over time. Popescu et al.^10^ were able to successfully determine the membrane fluctuations of a single RBC using DPM.^10^ Figure 6(e)^10^ shows the optical pathlength map of a single RBC with thickness profile along the direction of the arrow shown in Fig. 6(f).^10^ The nanometer membrane fluctuations over three points in the cell marked as 1, 2, and 3 are shown in Fig. 6(g) for a period of 10 s.^10^ It was observed that the fluctuations toward the edge of the cell, signified through the standard deviation, are more pronounced as compared to those at the center point of RBC. This remarkable measurement was possible due to the common path configuration of DPM, which provides nm sensitivity in optical pathlength measurements and the time resolution is small due to a single shot measurement capability of DPM.^10^

**Fig. 7:**
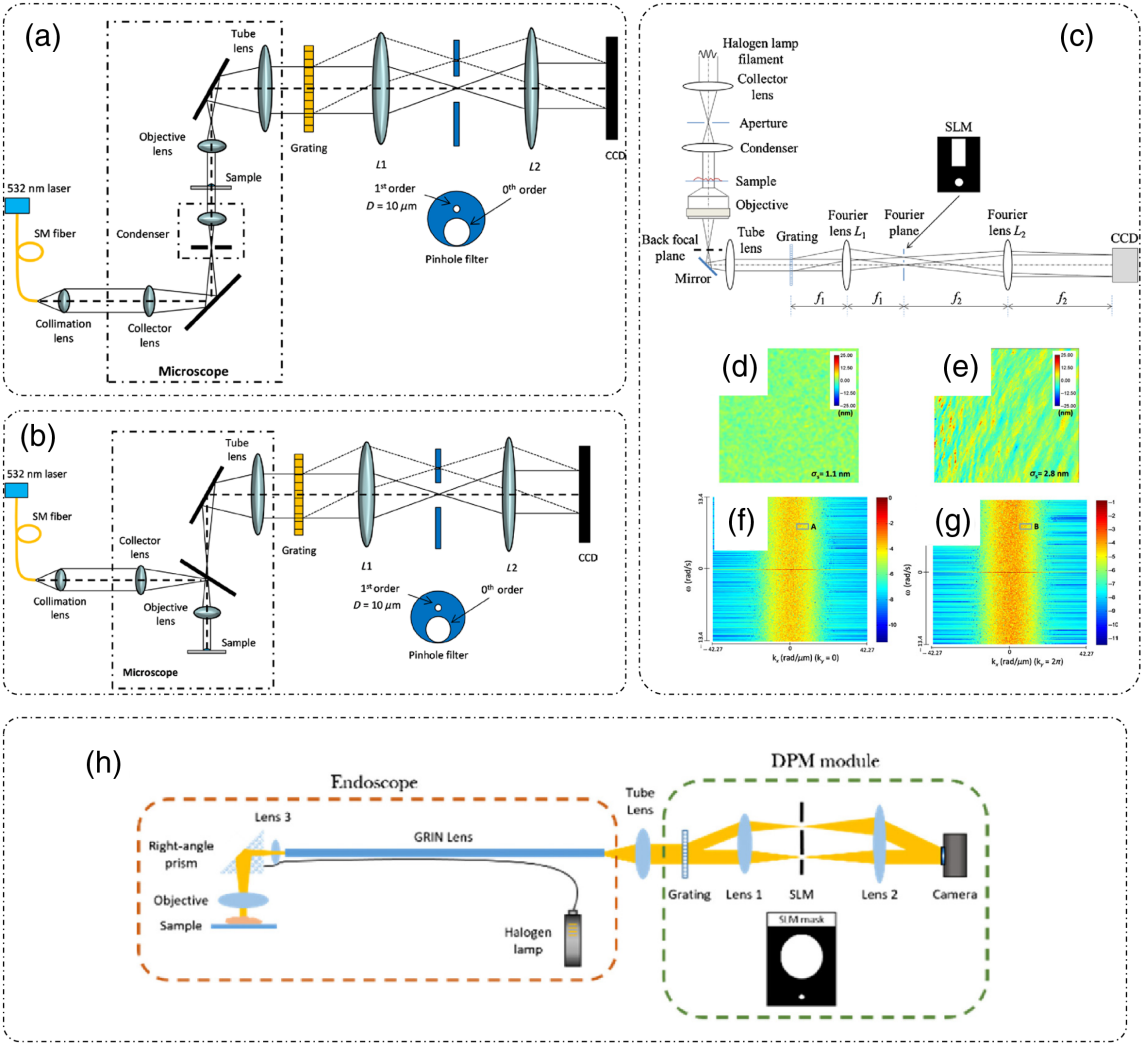
Geometries and variations of DPM: (a) transmission mode DPM (b); reflection mode DPM; (c) white light DPM (wDPM); (d) comparison of white light; and (e) laser DPM for sample-less images and the spatial frequency information (f) white light and (g) laser DPM, respectively; and (h) endoscopic DPM. (a), (b), (d), and (e) Reproduced with permission from Ref. 43, © 2014, Optica; (c), (f), and (g) reproduced with permission from Ref. 39, © 2012, Optica; and (h) reproduced with permission from Ref. 46, © 2018, Optica.

DPM can be implemented in both transmission and reflection modes. Figure 7(a) shows the transmission mode DPM where a 532 nm Nd:YAG laser is used as illumination source.^43^ The incident laser beam is passed through a single mode fiber to be coupled to a fiber collimation assembly that produces a collimated beam. This collimated beam passes through the collector lens in the microscope that focuses the beam into a point source at the condenser aperture. The condenser lens then transforms the point source into a collimated beam, fully illuminating the sample homogenously. Light after the sample is collected by the objective lens and relayed to the output port by the tube lens. The rest of the setup is as explained previously in this section.

The reflection mode geometry of DPM is as shown in Fig. 7(b).^43^ Instead of passing through the sample, light is now reflected or backscattered by it. The DPM setup remains the same as that in the transmission mode.^43^

Since these DPM systems employ a laser as an illumination source, they inherently suffer from a high-contrast speckle pattern across the field of view. The source of the speckle pattern is the high coherence of the laser radiation. In Refs. 39, 42, and 47, white light was used as an illumination source in both transmission and reflection geometry to eliminate the speckle in DPM images. White light DPM increases the spatial sensitivity of the DPM images by decreasing the noise threshold. Figure 7(c) shows the white light DPM (wDPM) setup, where light through a broadband source serves as an illumination beam.^39^ Creating a perfect plane wave reference beam is not possible with such a source. However, an approximation can be created by increasing the spatial coherence of illumination, by closing the condenser aperture. Due to the imperfect reference beam, the image formed at the camera plane suffers from halo artifact around the edges of the objects.^39^^,^^42^^,^^43^^,^^47^ Figures 7(d)–7(g) show the comparison between a sample-less area image obtained through wDPM and laser DPM, respectively.^39^^,^^43^

The spatial optical pathlength sensitivity is improved from 2.8 nm in laser DPM to 1.1 nm in wDPM. However, since white light is a broadband source, it is typically of low brightness and does suffer from dispersion, which can be reduced using achromatic optics and image processing to remove artifacts.^43^ Endoscopic DPM as shown in Fig. 7(h), is a very promising tool for *in vivo* diagnostics.^46^ It consists of an endoscope module constructed using a GRIN lens assembly and a standard DPM module.

DPM has been utilized for the study of various dynamic phenomena that include erythrocyte membrane fluctuations,^48^^,^^49^ which will be discussed in detail in Sec. 2.2.4.

##### Gradient light interference microscopy

Nonlinear (multiphoton) microscopy has been the traditional optical imaging method for thick/multicellular samples, which are multiple scattering in nature, such as embryos and organoids. Such nonlinear microscopy methods, however, involve high peak power illumination of the sample, which could result in damage to the sample by phototoxicity. GLIM, which is a low-coherence interferometry technique, was developed to counteract the problems associated with nonlinear microscopy and extract phase information from multiple scattering samples. The operating principle of GLIM is based on DIC, low-coherence interferometry, and holography.^11^ Due to broadband, incoherent illumination, and coherence gating phenomena, GLIM can achieve higher depth sectioning. The low-coherence interferometric aspect of GLIM and the resultant high-depth sectioning combined with phase-shifting reconstruction helps to suppress multiple scattering from the sample. Thus GLIM has been applied to imaging 3D samples for both structural as well as compositional information.^11^^,^^12^^,^^50^^,^^51^

**Fig. 8:**
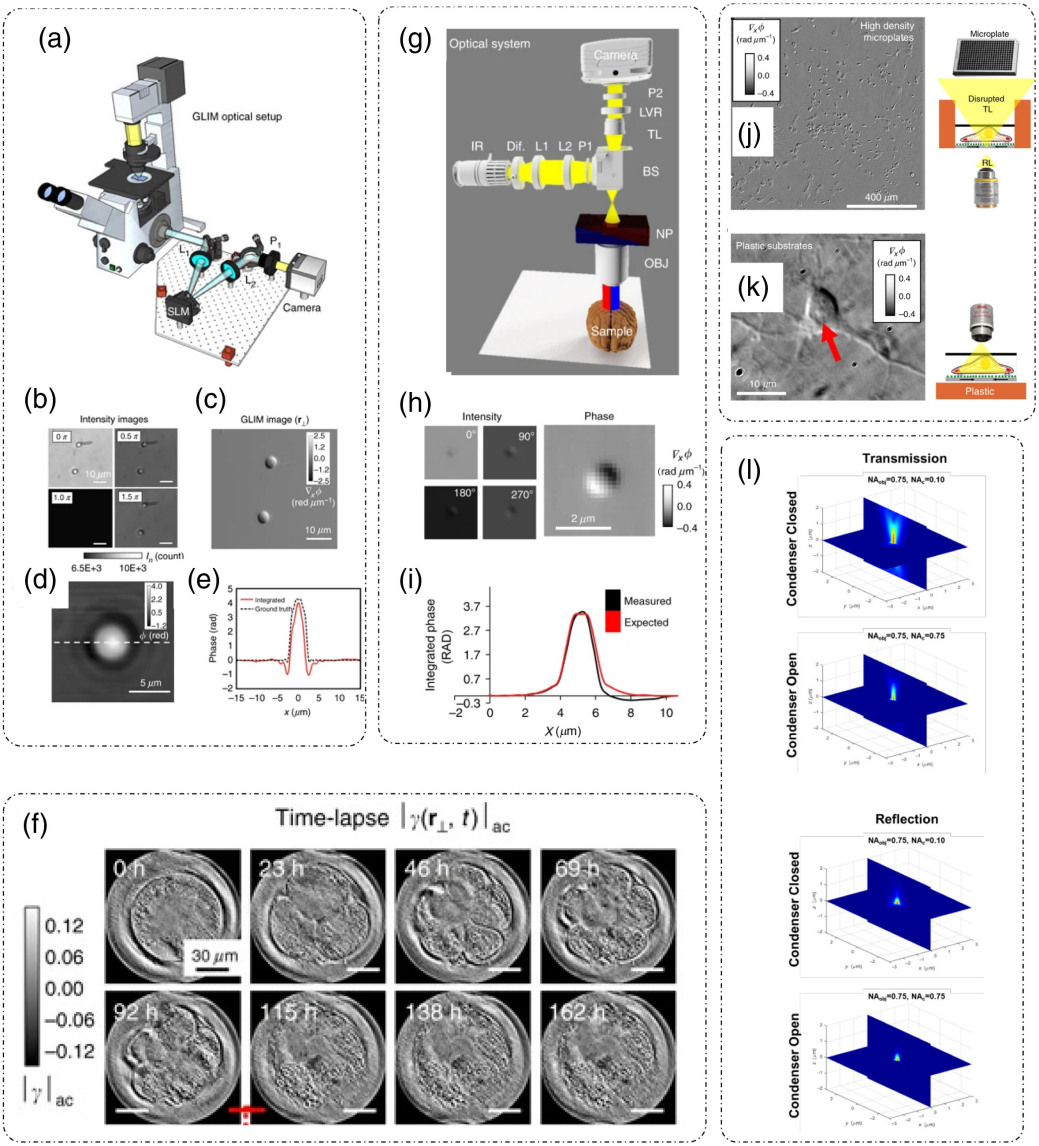
GLIM and epi-GLIM: (a) optical setup for GLIM; (b) four-phase shifted intensity detections; (c) phase gradient map extracted from four frames in (b); (d) phase map obtained after integration of (c) along shear direction; (e) comparison of measured and calculated phase distribution for the polystyrene bead; (f) time lapse imaging of Bovine embryo for a week performed using GLIM; (g) optical setup for epi-GLIM; (h) four-frames and extracted phase gradient image of a bead using epi-GLIM; (i) phase distribution comparison between calculated and measured backscattered phase for the polystyrene bead; (j) phase-gradient map of neurons in a high-density well-plate (1536 wells) imaged with epi-GLIM; (k) phase gradient map of a neuron imaged on a plastic substrate obtained through epi-GLIM; (l) comparison of axial psf in transmission and reflection GLIM geometries. (a)–(f) Reproduced from Ref. 11 under CC BY license; (g)–(l) reproduced from Ref. 12 under CC BY license.

GLIM has been developed as an add-on module for a standard DIC microscope [Fig. 8(a)].^11^ In a DIC microscope, the broadband (a broadband LED or a halogen lamp) illumination beam is divided by a Nomarski prism into two orthogonally polarized beams that are laterally sheared. The lateral separation is less than the width of the diffraction spot. After passing through the sample, these two beams carry similar image information but are phase shifted due to the lateral separation. A second Nomarski prism after the objective lens recombines the two beams. These two beams still cannot interfere due to having orthogonal polarizations. For interference to happen, in DIC operation, a polarizer is placed after the second Nomarski prism and is aligned at 45 deg to the polarizations of both the incoming beams. For GLIM operation, a liquid crystal variable retarder (LCVR) or an SLM that can introduce additional phase shifts $\phi_{n}=n\pi /2$, where n=0,1,2,3, to one of the beams [Fig. 8(b)^11^] is placed between the objective Nomarski prism and the final polarizer.^11^

The field at the camera plane is

$$U_{n}(r)=U(r)+U(r+\delta r)e^{i\phi_{n}}.$$ (23)

Here δr is the lateral shift between the two orthogonal polarization fields, and $\phi_{n}=n\pi /2$, n=0 to 3 is the phase modulation offset introduced by the LCVR, corresponding to the temporal modulation term ($\left\langle \omega \rangle (t-t_{r}\right)$) in Eq. (2). U(r) and U(r+δr) correspond to the two interfering fields in Eq. (2), representing object and reference fields. The intensity on the detector plane can then be expressed as

$$I_{n}(r)=I(r)+I(r+\delta r)+2|\gamma (r)|\cos[\phi (r+\delta r)+\phi_{n}-\phi (r)],$$ (24)

where $|\gamma (r)|=\sqrt{\left|I(r)||I(r+\delta r)\right|}=\langle U(r)U^{*}(r+\delta r)\rangle_{t}$ is the mutual intensity, which is the temporal cross correlation between the two laterally shifted, interfering beams at zero delay.^11^

The four-intensity frames, each captured with one of the four successive values of $\phi_{n}$, are then processed using the phase-shifting algorithm^35^ to extract phase gradient $\nabla_{r}\phi (r)=(\Delta \phi /\delta r)\delta \hat{r}=\tan^{-1}[\frac{I(x,y;3\pi /2)-I(x,y;\pi /2)}{I(x,y;0)-I(x,y;\pi )}]/\delta r.$ [Fig. 8(c)].^11^ Note that since GLIM is based on the DIC configuration, which generates an intensity image proportional to the gradient of the phase delay, the extracted quantity is a gradient. Whereas, in SLIM, where the four-frames provide phase delay [Eq. (20)] because the base modality is PC, which generates an intensity image proportional to the phase delay. The phase information is the integral of the gradient phase along the direction of shear [Figs. 8(d) and 8(e)]:^11^

$$\phi (r)=\int_{0}^{r}[\nabla_{r}\phi (r^{\prime })]dr^{\prime }+\phi (0),$$ (25)

where ϕ(0) is the initial /background phase. The risk of photodamage is extremely low due to the use of low-power density illumination and hence samples can be observed for longer durations. Nguyen et al.^11^ employed GLIM to study embryo growth and viability for a week [Fig. 8(f)].^11^

##### Epi-illumination gradient light interference microscopy

Thicker 3D objects, or objects placed on opaque substrates, are a limitation for transmission GLIM. Though nonlinear microscopy achieves better penetration depth due to larger wavelengths, it suffers from photodamage to the object eventually as illumination power requirements are high. To enable imaging of much thicker samples, GLIM was implemented in reflective mode by Kandel et al.^12^ This new QPI instrument, known as Epi-GLIM works on the same principle as GLIM. Figure 8(g) shows the optical setup for epi-GLIM.^12^ The base microscope in this case is a reflective, upright microscope configured in DIC mode. An Epi-GLIM module is attached to the output port of the microscope. Broadband illumination light is passed through a polarizer to make light incident on the Nomarski prism at 45 deg to its axis. Light is split into two laterally sheared, orthogonal polarization components by the Nomarski prism. Light reflected from the sample is combined into one beam with two orthogonal polarizations by the same Nomarski prism. This beam then enters the LCVR and is modulated in steps of π/2. An analyzer placed right before the camera at 45 deg to the incoming polarizations causes interference to happen at the camera plane. The reconstruction principle is the same as described in transmission GLIM.

Figures 8(h)–8(i) show the phase reconstruction of a 1.9  μm polystyrene bead immersed in oil and placed on a reflective surface.^12^ It is to be noted that since the object is placed on a reflective surface the expected phase value is twice when the same sample is placed on glass and measured through transmission GLIM.^12^

Another advantage of Epi-GLIM is that it can be used to image high-density well plates, in which the well size is smaller because of large number of wells. Liquid media form a meniscus at the edge of these wells, which causes the illumination to be nonuniform, and thus transmission imaging is not well suited for such plates.^12^ Figure 8(j) shows an Epi-GLIM image of one field of view from a 1536 well plate.^12^ The background shows no inhomogeneity. Transmission DIC is not recommended when using a plastic bottom plate, which is birefringent and thus degrades the DIC operation. However, Epi-GLIM overcomes this problem as the measured signal is reflected from and not passing through the plastic. Figure 8(k) shows a neuron image cultured on a plastic substrate and measured through Epi-GLIM.^12^

Since in reflection geometry the objective also acts as a condenser, it helps in increasing the frequency coverage of Epi-GLIM system as compared to the transmission geometry. A double pass through the objective effectively increases the NA of the system.^12^ The improvement in the axial point spread function (psf) can be seen in Fig. 8(l).^12^

##### Digital holographic microscopy

DHM is a computed imaging technique based on the optical holography that seeks to numerically reconstruct the complex-valued field. An interferogram is formed at the hologram plane by interference between the object and reference wave from which complex field is extracted. The extracted field is then numerically propagated to different axial locations by the use of wave propagation equations.^14^

**Fig. 9:**
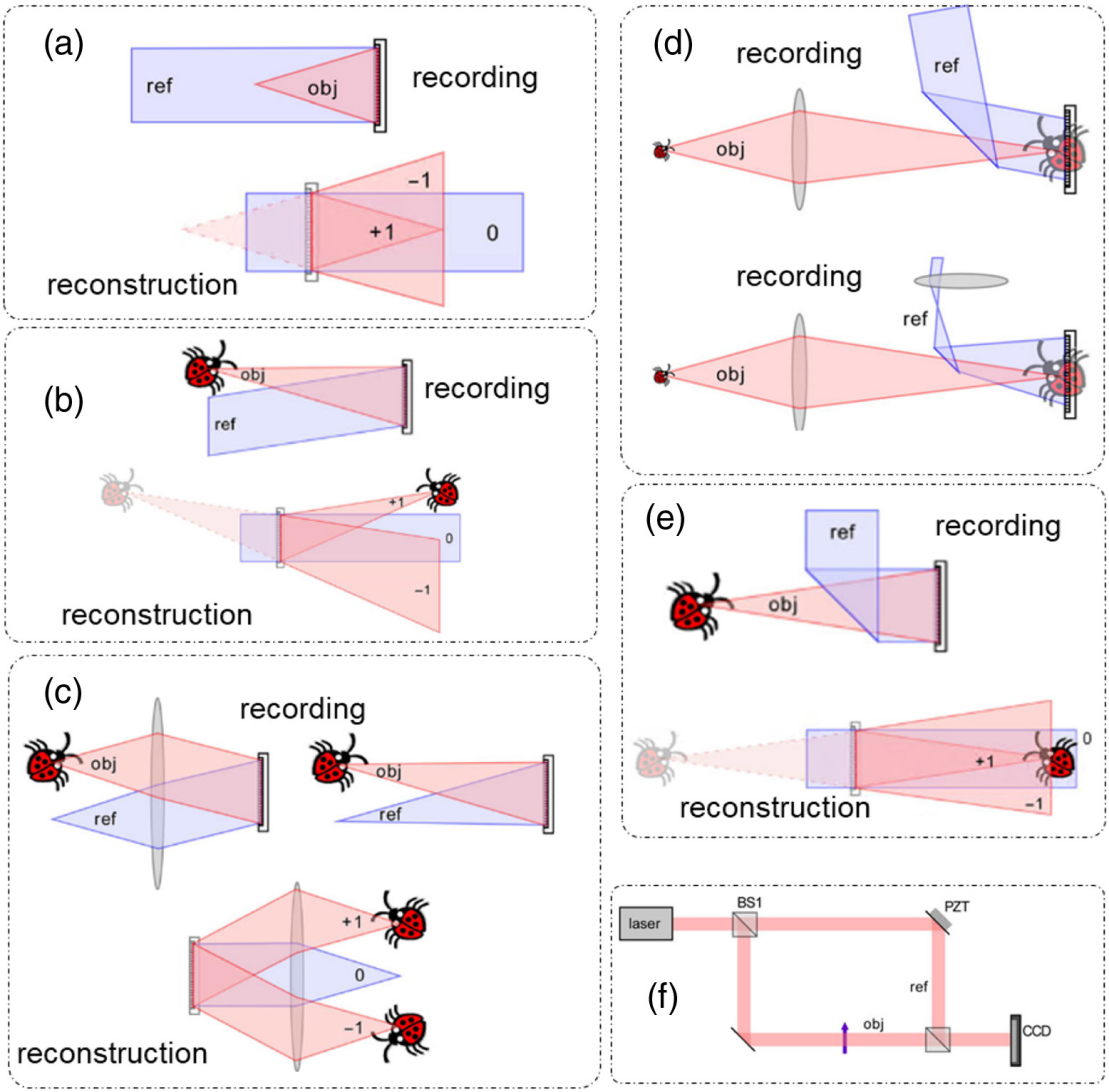
Configurations of digital holography: (a) Gabor holography; (b) off-axis Fresnel holography; (c) Fourier transform holography; (d) image plane holography; (e) inline holography; and (f) phase-shifting holography. (a)–(f) Reproduced with permission from Ref. 14, © 2010, SPIE.

Figure 9 shows different configurations of digital holography as discussed in detail in Ref. 14. In Gabor holography, both reference and object beams are extracted from the same incoming beam based on the weakly scattering property of the sample [Fig. 9(a)].^14^ The portion of the incident light scattered from the object becomes the object beam and the unscattered portion becomes the reference beam.^14^ In off-axis Fresnel holography [Fig. 9(b)],^14^^,^^52^ the reference beam is an off-axis plane wave. As explained previously in the discussion of DPM, for phase recovery, one of the diffraction orders is extracted from the Fourier transform of the hologram.^53^ The Fresnel transform (based on the distance between the object and the hologram plane) is applied for the numerical reconstruction of complex fields from the measured hologram.^14^ In Fourier transform holographic microscopy, the reference is a point source placed at the object plane. To reconstruct the image, only one Fourier transform is required [Fig. 9(c)].^14^^,^^54^^,^^55^ For a lens-based Fourier holography setup, the reference is a plane wave and the object and the hologram plane are both at a focal length away from the Fourier transform lens.^14^ Anand et al.^56^ discussed another geometry for Fourier transform holography where a single beam is divided into reference and object beams using an image plane aperture. In this configuration, the pinhole samples a portion of the object beam, which acts as a reference point source. The object beam is created by passing through a diffuser (placed at the same plane as the pinhole). These beams then form a hologram at the CCD plane.

Another holography configuration is image plane holography,^57^ which requires the object to be placed very close to the hologram plane.^14^ Figure 9(d)^14^ illustrates one example where the object for the hologram is a magnified object image projected at the hologram plane^57^ or in another configuration the reference beam is also passed through another lens to magnify the hologram. This configuration is suitable for low-coherence interferometric techniques.^14^ For in-line holography [Fig. 9(e)], both the object and reference beams are coincident.^14^ Due to this coincidence, twin images [the intended (real) image and the unwanted, out-of-focus, complex conjugate image of the real image] and strong DC components occur because of the presence of incident reference wave. Image processing techniques are used to remove these unwanted artifacts. The in-line geometry, however, provides an increase in resolution as required for biological applications.^14^^,^^58^ Phase-shifting digital holography [Fig. 9(f)]^14^^,^^59^ replaces the use of Fourier transform of a single hologram to retrieve complex field by phase-shifting reconstruction using multiple holograms (discussed in SLIM and GLIM). The phase of the reference beam is modulated in steps of π/2 to generate four holograms.^59^^,^^60^ The complex field is retrieved at the hologram plane using phase-shifting reconstruction (discussed previously in the presentation of SLIM/GLIM). This field can then be numerically propagated along the propagation direction. Phase-shifting digital holography has also been reported with two-step phase-shifting^61^^–^^63^ or three-step phase-shifting and reconstruction.^64^ Phase-shifting reconstruction eliminates the artifacts of DC and, first-order twin images, conditioned upon accurate phase shifting.^14^^,^^59^^,^^65^^–^^71^

The reconstructed image in DHM can suffer from phase aberrations due to imaging optics. A large amount of research has been devoted to numerically compensating for or eliminating the aberrations using methods, such as using a numerical lens,^72^^,^^73^ conjugated holograms,^74^ principal component analysis (PCA) of phase map to extract aberrations,^75^ nonlinear optimization to minimize phase variations,^76^ geometric transformation-based aberration correction,^77^ least square fitting and segmentation,^78^ and wavefront correction at the hologram plane.^79^ Aberration correction through deep learning is addressed in Ref. 80, where a convolutional neural network can estimate the background phase, and aberration is then corrected using Zernike polynomial fitting. Kim^14^ and Marquet et al.^71^ provided a useful review of different digital holographic techniques and numerical methods employed for reconstruction and aberration correction.

**Fig. 10:**
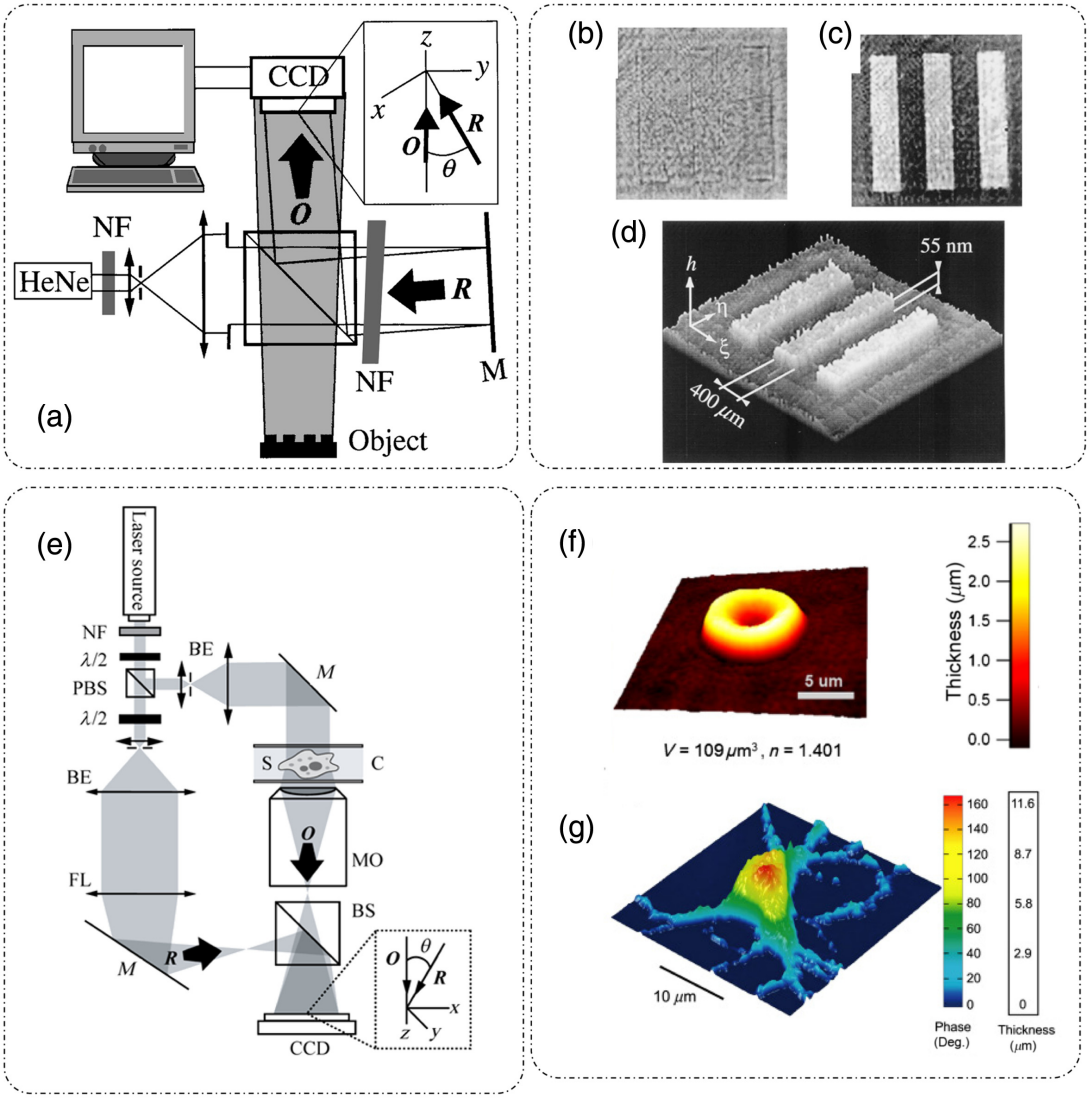
DHM: (a) reflection mode geometry. Reflection mode DHM measurements of a pure phase object: (b) amplitude; (c) phase; and (d) 3D height distribution; (e) transmission mode geometry; (f) thickness profile of RBC; (g) phase and thickness maps of a mouse cortical neuron measured through transmission DHM. (a)–(d) Reproduced with permission from Ref. 52, © 1999, Optica; (e)–(g) reproduced from Ref. 71 under CC BY license.

QPI based on DHM principles has been employed extensively in biomedical applications.^58^^,^^71^^,^^81^^,^^82^ Figures 10(a) and 10(e) show one representative optical setup for each reflection and transmission DHM geometry.^52^^,^^71^ In the reflection geometry illustrated in Fig. 10(a), light from a coherent source (a HeNe laser, 632.8 nm) is spatially filtered (using two lenses and a pinhole in a beam expander geometry) and a half of the beam illuminates the object upon reflection through a beam splitter and is back scattered from the object (O). The other half (R) is transmitted by the beam splitter onto a mirror (M), which tilts the beam at an off-axis angle with respect to the object beam. The tilted reference beam is then reflected toward the CCD plane where it interferes with the backscattered object field and forms the hologram, which can then be numerically reconstructed and propagated at different axial locations.^52^ Figures 10(b)–10(d) show the images of fabricated phase object measured through reflection DHM.^52^ Transmission DHM is shown in Fig. 10(e),^71^ where a collimated coherent source is focused onto the sample by condenser lens. The other half of the collimated illumination beam is reflected by the beam splitter onto a mirror that reflects the collimated beam at an angle toward the CCD plane. The field scattered from the sample is collected by the objective lens and is relayed to the CCD plane as the object beam. The hologram from the interference of object and reference fields is formed at the CCD plane, which can then be reconstructed using techniques discussed above. The thickness profile of an RBC and phase and thickness profile of a mouse cortical neuron measured through transmission DHM are as shown in Figs. 10(f) and 10(g), respectively.^52^^,^^71^

We consider the illumination, sample, objective lens and CCD configuration as depicted in Fig. 10(e), where the sample is illuminated with a plane wave $U_{0}$ with incident wavevector $k_{i}$. The sample is placed at a distance $d_{s}$ from the objective lens (of focus $f<d_{s}$). The image plane is at a distance $d_{i}$ from the objective lens, where the field is $U_{1}$. CCD is positioned in between the objective lens and image plane at a distance d from the image plane. Following the mathematical formulation in Ref. 3, the field at the image plane can be expressed as

$$U_{1}(x,y)=\frac{1}{\left|M\right|}U_{0}(\frac{x}{M},\frac{y}{M}),$$ (26)

where the field is a magnified version of the object with magnification $M=\frac{d_{i}}{d_{s}}$. This field can be back propagated to the CCD plane using Fresnel propagation kernel in the reverse direction as

$$U_{D}\left(x,y\right)=U_{1}\left(x,y\right){Ⓥ}_{\left(x,y\right)}e^{-i\frac{\beta_{0}}{2d}(x^{2}+y^{2})}.$$ (27)

This field interferes with the off-axis reference field $U_{r}$ incident with wavevector $k_{r}$ [to introduce spatial phase modulations as indicated by the second term of cosine [$(\langle k\rangle -k_{r})\cdot r$] in Eq. (2)], assumed to be a plane wave here, and the resultant hologram is

$$U_{H}(x,y)=U_{D}(x,y)+|U_{r}|e^{ik_{r}\cdot r}.$$ (28)

This detected hologram is then Fourier transformed, and the complex field is retrieved by spatial filtering where the signal corresponding to one of the sinusoidal peaks is selected and the rest (DC and the twin image) is filtered out. The 2D Fourier transform of the resultant intensity gives

$${\overset{˜}{I}}_{H}(k_{x},k_{y})={\overset{˜}{I}}_{0}(k_{x},k_{y})+{\overset{˜}{I}}_{+1}(k_{x},k_{y})+{\overset{˜}{I}}_{-1}(k_{x},k_{y}),$$ (29)

where

$${\overset{˜}{I}}_{0}(k_{x},k_{y})=I[|U_{D}(x,y){|}^{2}+|U_{r}{|}^{2}],\;{\overset{˜}{I}}_{+1}(k_{x},k_{y})=|U_{r}|{\overset{˜}{U}}_{D}(k_{x}-k_{rx},k_{y}),\;{\overset{˜}{I}}_{-1}(k_{x},k_{y})=|U_{r}|{\overset{˜}{U}}_{D}(k_{x}+k_{rx},k_{y}).$$ (30)

Here I denotes the 2D spatial Fourier transform, and $k_{rx}$ is the reference wavevector incident along x direction.

After removing the DC and twin image, shifting the first-order term back to origin and taking the inverse Fourier transform, the resultant object field can be recovered as

$$U_{1}(x,y)\propto I^{-1}[{\overset{˜}{I}}_{+1}(k_{x}+k_{rx},k_{y}).e^{-i\frac{d}{2\beta_{0}}(k_{x}^{2}+k_{y}^{2})}].$$ (31)

Now, this field can be axially propagated over any distance through either Fresnel propagation or Huygens’s convolution with the Green’s function.

DHM based on a backscattering geometry claims to have axial sensitivity/accuracy of 0.9 nm and temporal stability of 0.8 nm^71^^,^^83^ in a dual-wavelength reflection mode instrument. To remove the object dependent noise, interwavelength noise and shot noise, different averaging schemes have been employed as described in Ref. 83.

**Fig. 11:**
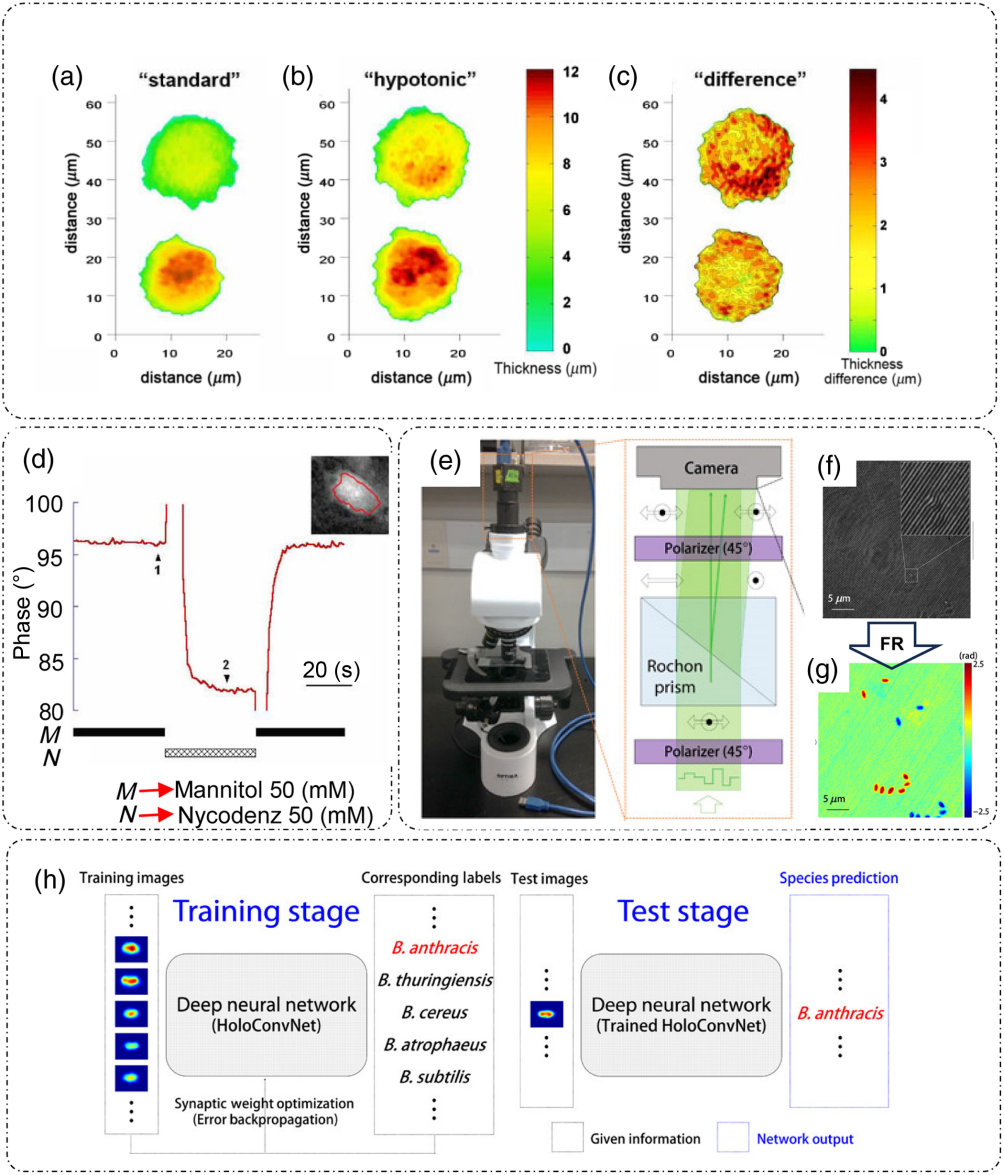
Applications of DHM in Single cell dynamics-Measurement of thickness variations of neuron cell body in (a) standard conditions (before); (b) Hypotonic conditions (3 minutes after the onset of a hypotonic shock); (c) difference between (b) and (a). (d) refractive-index and thickness decoupling: temporal phase profiles with solid rectangles at bottom showing durations with standard solution and dashed rectangles showing durations with decoupling solution, for refractive index decoupling with different immersion media; (e) QPIU; (f) raw holographic data; (g) extracted phase profile; and (h) machine learning application based on holographic phase data in (g) to detect *Bacillus anthracis*. FR, field retrieval. (a)–(d) Reproduced with permission from Ref. 84, © 2005, Optica; (e)–(h) reproduced with permission from Ref. 85 under CC-BY license, with some rights reserved; reprinted with permission from AAAS.

The high spatial sensitivity and low acquisition time—of the order of tens of μs—enables DHM to visualize and quantify the dynamic processes occurring at single cell level. As an example,^84^ Figs. 11(a)–11(c) reveal the changes induced in single neuron cell bodies under hypotonic stress, with the left panel showing the thickness maps before stress [Fig. 11(a)], middle panel after stress [Fig. 11(b)], and right panel [Fig. 11(c)] showing the difference phase map between normal and stressed cell bodies.^84^ Thickness changes and refractive index fluctuations are decoupled using a procedure that involves measurement of holograms with varying refractive index of the surrounding media.^84^ The corresponding phase changes are shown in Fig. 11(d) with the phase measurements averaged over the surface of cell shown in the inset with boundaries marked in red.^84^ The phase measured at point 1 can be expressed as^84^

$$\phi_{1i}=\beta_{0}({\overset{¯}{n}}_{i}-n_{s})d_{ci},$$ (32)

where $\phi_{1i}$ is the phase at i’th spatial location, $\beta_{0}=2\pi /\lambda$ is the free-space wavenumber, ${\overset{¯}{n}}_{i}$ is the axial projection of refractive index of cell at i’th spatial location, $n_{s}$ is the refractive index of the surrounding media at point 1, and $d_{ci}$ is the thickness of cell at i’th spatial location.

After replacing the immersion media with another one, having a different refractive index, the phase at point 2 can be expressed as^84^

$$\phi_{2i}=\beta_{0}({\overset{¯}{n}}_{i}-[n_{s}+\Delta n])d_{ci}=\phi_{1i}-\beta_{0}\Delta nd_{ci},$$ (33)

where Δn is the change in refractive index between the two solutions. Solving Eqs. (32) and (33) for ${\overset{¯}{n}}_{i}$ and $d_{ci}$ yields^84^

$$d_{ci}=\frac{\phi_{1i}-\phi_{2i}}{\beta_{0}\Delta n}$$ (34)

and

$$n_{ci}=n_{s}+\frac{\Delta n\phi_{1i}}{\phi_{1i}-\phi_{2i}}.$$ (35)

Hence, the measured optical phase map can be decoupled into refractive index and thickness map by immersing the sample in two different media.^71^^,^^84^^,^^86^ Other decoupling procedures involve assumptions, such as the spherical structure of cell, or involve measurements at two different wavelengths.^86^^,^^87^ An extensive review of refractive index decoupling techniques can be found in Ref. 86.

**Fig. 12:**
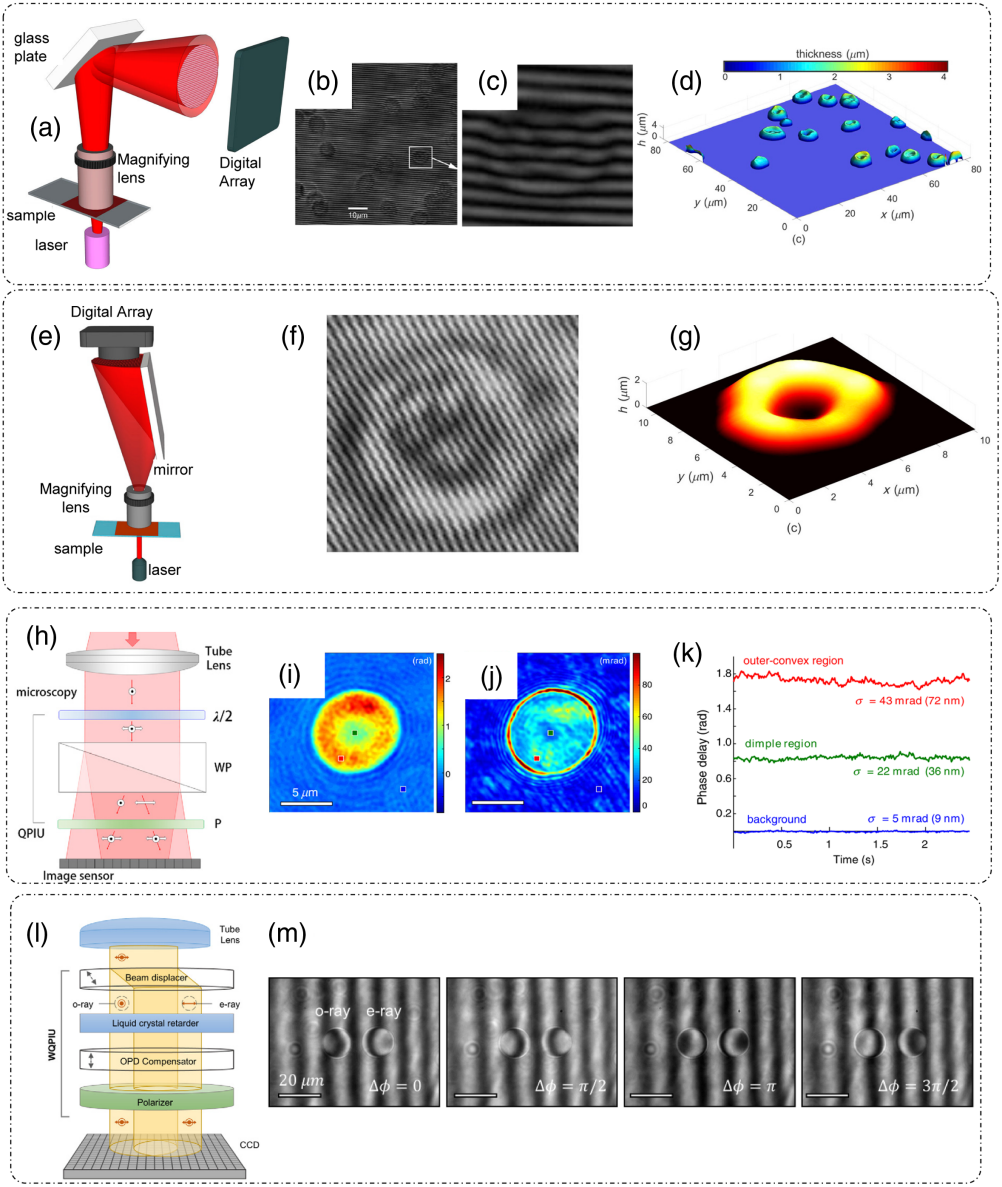
Self-referencing DHM: (a) lateral shearing DHM; (b) recorded hologram; (c) zoomed-in view of the white box in (b); (d) thickness profile of RBC measured through (a); (e) wavefront division DHM; (f) recorded hologram; (g) thickness profile of RBC measured through (e); (h) Wollaston-prism based polarization-splitting DHM with corresponding measurements of RBC; (i) mean phase map; (j) characterization of mean phase fluctuations in terms of standard deviation; (k) temporal phase fluctuations in various parts of RBC [indicated by dots in Figs. (i) and (j)] as compared to background; (l) beam displacer-based white-light DHM; and (m) corresponding four-phase shifted interferograms. (a)–(g) Reproduced from Ref. 88 under CC BY license, with subfigure (a) adapted with permission from Ref. 99, © 2012, Optica, and Ref. 100, © 2017, Optica; and subfigure (e) adapted with permission from Ref. 101, © 2012, Optica. (h)–(k) Reproduced with permission from Ref. 90, © 2014, Optica. (l), (m) Reproduced with permission from Ref. 91, © 2016, Optica.

Self-referencing DHM is an important mode of DHM that can be implemented in both in-line or off-axis configurations.^88^^,^^89^ In this type of holography, a single object beam is divided into object and reference beam like in DPM, which then interfere to form the hologram. Division of a single beam can be obtained either by splitting the object beam on the basis of amplitude,^89^ polarization,^90^^,^^91^ by providing a lateral shear^88^ etc., or through division of wavefront^88^^,^^92^ as shown in Figs. 12(a) and 12(e).^88^ Figures 12(b)–12(d) and Figs. 12(f)–12(g) show the corresponding holograms and reconstructed thickness maps for RBCs for the lateral shearing and wavefront division, respectively.^88^ An example of a QPI technique based on the polarization splitting is shown in Fig. 12(h) and Fig. 12(l).^90^^,^^91^ Through this instrument called a quantitative phase imaging unit (QPIU),^90^^,^^91^ phase information can be extracted from the recorded hologram provided the two sheared object and reference beams form nonoverlapping sample images at the detector plane.^90^^,^^91^ Quantitative mean phase measurements of an RBC are shown in Figs. 12(i)–12(k), with Fig. 12(i) shows the mean phase map, Fig. 12(j) shows the height fluctuations represented by standard deviation map, and Fig. 12(k) shows the temporal fluctuations of height of the cell, all measured through QPIU.^90^ Figure 12(m) shows the four-phase-shifted interferograms acquired for phase extraction.^91^ Anand et al.^88^ provided an informative review of self-referencing DHM techniques. Recently, Kumar et al.^93^ introduced a common-path reflective mode off-axis DHM based on the division of the illumination beam into object (90% of incidence beam) and reference (10% of incidence beam) beams using a plate beam splitter with reportedly increased field-of-view.

QPI through DHM has been used for the identification and characterization of diseases by studying the refractive index distribution of healthy and diseased cells.^94^ Cell sorting or classification of cells using quantitative phase data obtained through DHM and data processing techniques has been discussed in Ref. 95. Phase imaging can be very useful for studying erythrocytes, as the decoupling of refractive index and thickness from the phase measurements is easy due to the anucleate structure of these cells. There are several studies involving RBCs in the QPI field, such as differentiation of mature and immature RBCs based on the phase measurements,^96^ measurements of cell membrane dynamics,^97^ etc. There are also studies that involve observation and quantification of neuron activity by monitoring water transport through membranes and its effect on phase.^71^^,^^97^ DHM has also been used for the disease detection and quantification of corresponding pathophysiological effects, i.e., in sickle cell disease.^98^

Algorithms for numerical reconstruction, aberration correction, and image analysis have been developed simultaneously for DHM. Machine learning is another research direction that is quickly being incorporated in the microscopy regime. As an example, for automatically differentiating between RBCs from two different organisms and diagnosis of sickle cell disease in patients, machine learning algorithms were employed in Ref. 102. That study demonstrated high accuracy in both classification tasks. Kim,^14^ Xu et al.,^58^ Marquet et al.,^71^ Lee et al.,^81^ Dardikman and N. T. Shaked,^86^ and Kemper et al.^103^ reported more examples of biological applications of DHM. Another important example relates to biowarfare security.^85^ In the study of Jo et al.,^85^ holographic data [Figs. 11(f) and 11(g)] obtained using QPIU^90^^,^^91^ [Fig. 11(e)] were combined with deep learning to train a network to detect and classify *Bacillus anthracis* spores from other cells within the Bacillus species as shown in Fig. 11(h).^85^

##### Hilbert phase microscopy

**Fig. 13:**
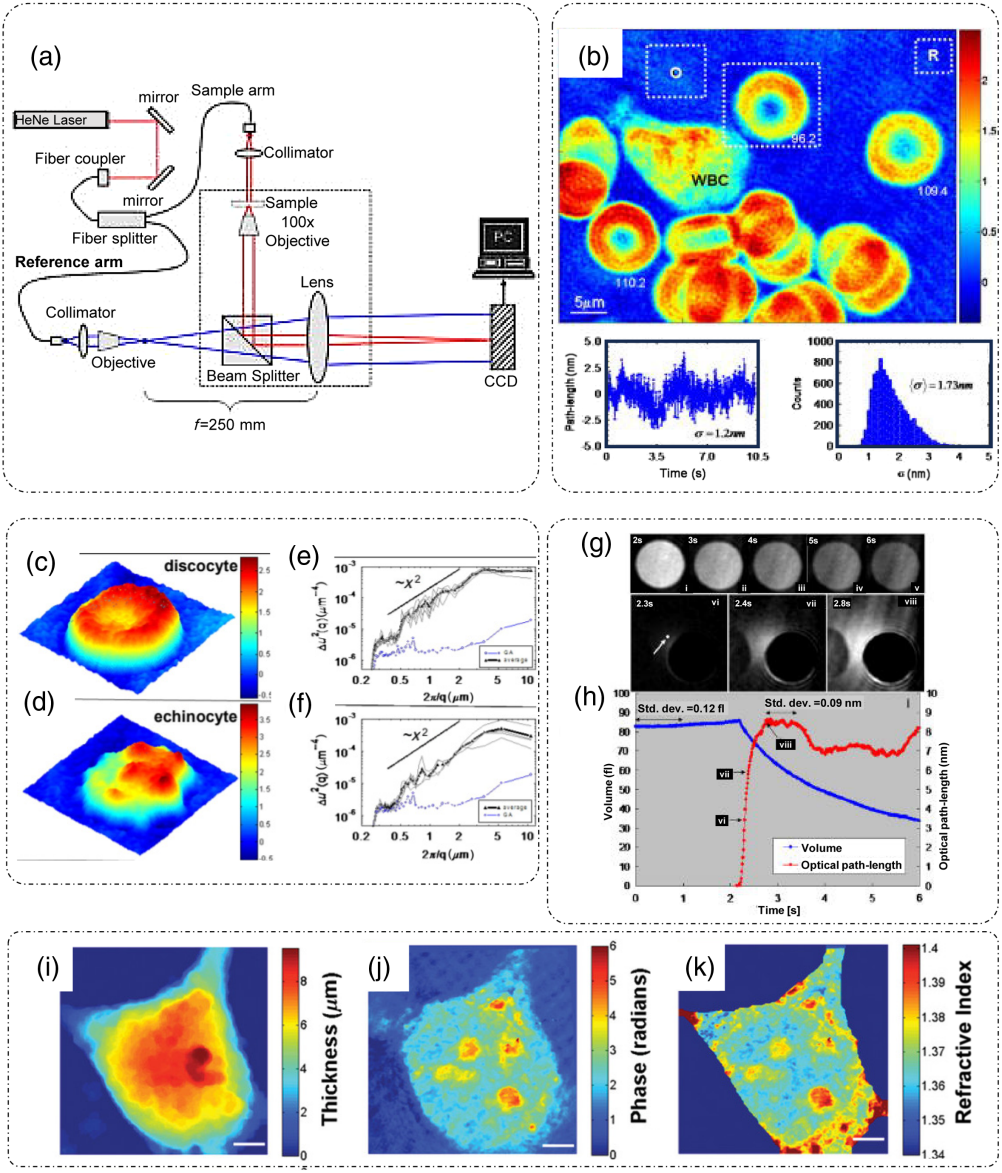
HPM: (a) setup and (b) phase measurements of whole blood smear with temporal and spatial optical pathlength sensitivity measurements corresponding to one point and all points respectively for the region inside the square marked O in the phase map. Cell topography and membrane MSD curves for (c), (e) discocyte and (d), (f) echinocyte. (g) Cellular lysis, volumetric fluctuations, and optical pathlength fluctuations measurements. Refractive index decoupling: (i) contour image and thickness measurements of a HeLa cell from reflectance confocal microscopy, (j) phase measurements of the same cell in (i) from HPM, and (k) decoupled refractive index distribution of the same cell as in (i). (a), (b), (g), and (h) Reproduced with permission from Ref. 104, © 2005, SPIE; (c)–(f) reproduced with permission from Ref. 105, © 2006, Optica; and (i)–(k) reproduced with permission from Ref. 106, © 2009, American Chemical Society.

HPM is a QPI technique that is based on off-axis digital holography.^13^ The optical setup is as shown in Fig. 13(a).^104^ Light from the laser (HeNe, 632.8 nm) is coupled to a single-mode fiber and fed into a 1×2 fiber splitter that splits the beam into two paths. The object beam is collimated and passes through the sample, collected by the objective lens, and imaged onto the CCD plane by the tube lens. The other half of the original beam traverses a separate path where it is collimated and focused by a lens to a point source, which is then Fourier transformed by the tube lens to form a plane wave at the CCD plane. This beam, which is slightly tilted with respect to the optical axis, becomes the reference beam for forming the interferogram.

The interferometric image captured by the CCD can be expressed as^3^^,^^13^

$$I(r_{⊥})=I_{0}+I_{1}(r_{⊥})+2\sqrt{I_{0}I_{1}(r_{⊥})}\;\cos[\alpha x+\phi (r_{⊥})],$$ (36)

where $I_{0}$ is the reference irradiance, $I_{1}$ is the object irradiance, $r_{⊥}=(x,y)$, and α is the spatial modulation frequency defined by the angular offset between the reference and object beams. In Eq. (36), $\phi (r_{⊥})$ is the quantity of interest, i.e., the spatially dependent phase perturbation induced by the object. The Fourier transform of this interferogram contains three peaks, one represents zero-frequency information, and the other two are peaks corresponding to the cosine term. To extract the cosine term, the interferogram is high-pass filtered. This cosine term represents the real part of the complex analytic signal $e^{i[\alpha x+\phi (r_{⊥})]}$. The imaginary part of the exponent is related by Kramers–Kronig relationship and can be calculated from the real part using Hilbert transform as^3^^,^^13^

Im(ei[αx+ϕ(r⊥)])=1πP∫−∞∞Re(ei[αx′+ϕ(r⊥)])x−x′dx′. (37)

After the extraction of full complex analytic signal, the desired phase term can be retrieved as φ=[αx+ϕ(r⊥)]=tan−1(Im(ei[αx+ϕ(r⊥)])Re(ei[αx+ϕ(r⊥)])), and the phase information from the object can be calculated as $\phi (r_{⊥})=\varphi -\alpha x$.^13^^,^^104^ Since HPM only requires a single-shot measurement, the speed of acquisition is remarkably high and is only limited by the camera frame rate. This high acquisition speed (ms) and quantitative measurements of optical pathlengths (nm) make HPM suitable for fast dynamic observations, such as membrane dynamics.^104^ As discussed earlier, decoupling of thickness and refractive index is quite easy in case of RBCs because of their anucleate structure, which makes the cell just a homogenous solution of hemoglobin. Figure 13(b) shows the phase map of a collection of RBCs placed between two thin glass coverslips.^104^ The temporal and spatial sensitivities of HPM were reported to be 1.2 and 1.73 nm, respectively.^104^ Note that this is not a common path instrument and to improve the temporal stability another HPM geometry was reported in Ref. 105, where a stabilization loop-based negative feedback corrects for the vibration induced orientation shifts of the interfering beams. The ability of HPM to quantify cell membrane fluctuations was shown in Ref. 105, where the cell topography for two types of RBCs (normal discocyte and spiculated echinocyte) is shown in Figs. 13(c) and 13(d). The time-lapse phase images of the two cell types were acquired for 1000 instances with a delay of 10.3 ms between two consequent acquisitions. The mean squared displacement (MSD) was calculated and Figs. 13(e) and 13(f) show the inverse square dependence of MSD on wavenumber q.^105^ These results hint toward the existence of a deterministic component in the membrane fluctuations, which was otherwise supposed to be of purely thermal (and hence random) in origin because of the lack of such nanometer precision measurement techniques.^3^^,^^105^

In addition to membrane dynamics, many other cellular phenomena like lysis can be observed and quantified by HPM. An example of an RBC undergoing lysis acquired through HPM is shown in Fig. 13(g), and the associated changes in the volume of the cell calculated through the thickness measurements are indicated by the blue curve in Fig. 13(h).^104^ This curve shows that during lysis, the volume of a cell decreases as it empties its hemoglobin content to the surrounding media. The optical pathlength increase of a neighboring point is also shown in the same plot indicated with red curve, which shows that the optical pathlength of the point is increasing due to increased hemoglobin release from the cell over time.^104^

Simultaneous thickness measurements from the contour maps [Fig. 13(i)] and phase [Fig. 13(j)] from a reflectance confocal and HPM system, respectively,^106^ are shown to aid the decoupling process and consequently the pure axially averaged refractive index map [Fig. 13(k)] of any cell can be extracted as discussed in Ref. 106.

HPM has also been applied for studying scattering properties of tissues.^107^ When the slice thickness of tissue slices is known, one can infer the axially averaged refractive index from the phase maps. Using HPM, it was shown that the axially averaged refractive index distribution varies between the organs from which the tissue slice originated, with the brain having the lowest mean of distribution and liver, the highest.^107^

#### 2.2.4 Fourier transform light scattering

Light scattering measurements are divided into two categories: ELS (discussed here) and quasielastic or dynamic light scattering (DLS) (discussed in part 2 of this review).^15^ Because they can inform refractive index estimation, elastic light scattering measurements provide information about the structure of the static objects under study. On other hand, DLS measurements can provide information about the temporal dynamics of the sample because they are sensitive to the diffusion coefficient of an ensemble of particles.^15^ Current methods that enable light scattering measurements suffer from either low spatial resolution by estimating the scattering properties based on a group of particles or low throughput in case of particle tracking measurements.^15^

FTLS is a technique that combines the high spatial resolution of image plane measurements in optical microscopy and intrinsic averaging of scattering measurements to measure elastic and DLS by biological samples.^15^ It is a spatial domain equivalent of Fourier-transform infrared spectroscopy since all possible scattering frequencies are measured at each spatial location.^3^

The complex field is measured at the image plane using DPM (for details see “Diffraction phase microscopy” section) and is then propagated to the Fourier or scattering plane to determine the scattered intensity distribution.^15^

Following the reconstruction process in DPM as discussed previously in “Diffraction phase microscopy” section, the complex field U(r,t) comprising of amplitude and phase is extracted from the single interferometric intensity measurement. Propagation of this extracted, complex field to the scattering plane can be realized through a spatial Fourier transformation:^3^

$$\overset{˜}{U}(q,t)=\int U(r,t)e^{-iq\cdot r}d^{2}r,$$ (38)

where the 2D integration is performed over the field of view.

**Fig. 14:**
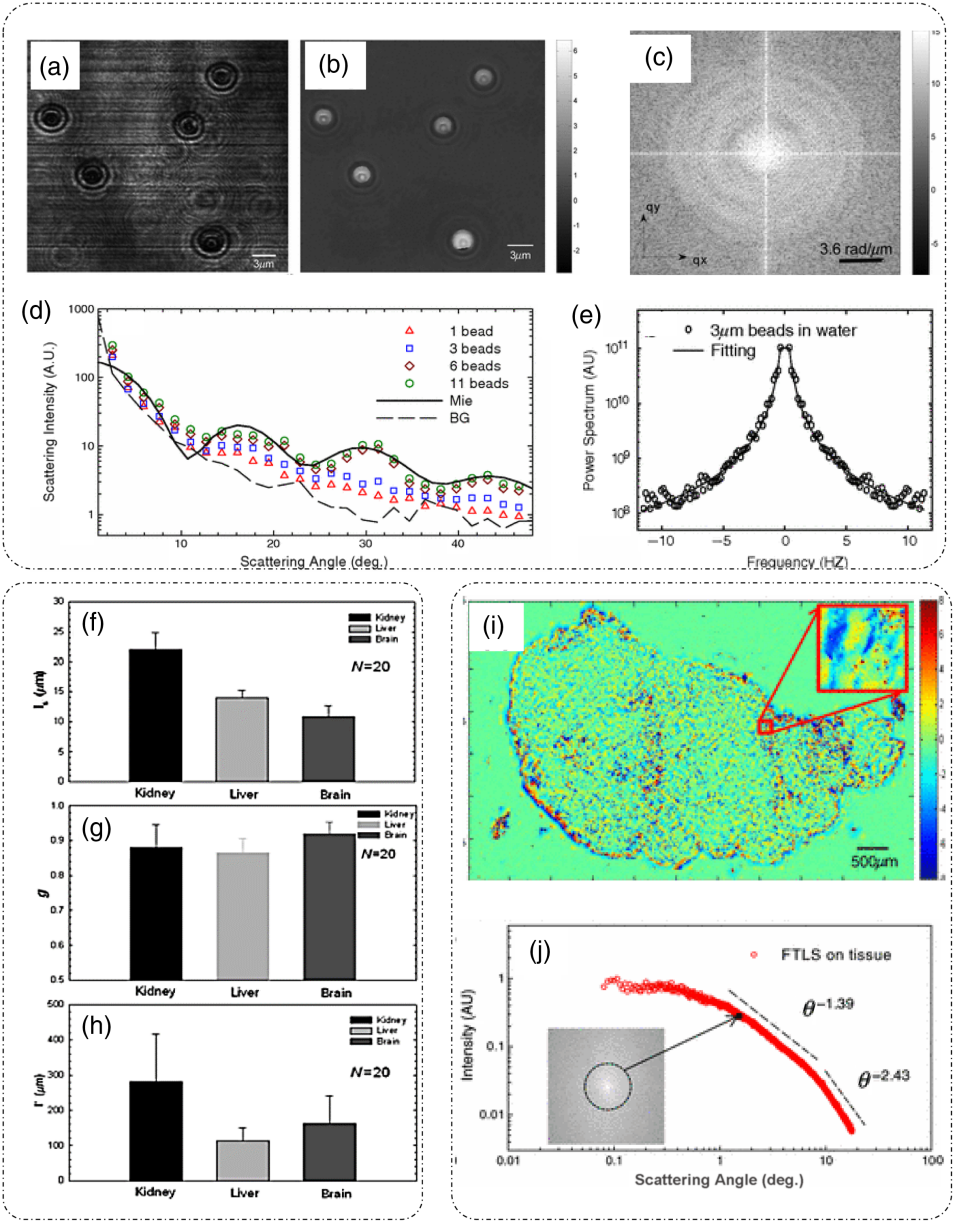
Fourier transform light scattering (FTLS): (a) amplitude measurement; (b) phase measurement; (c) spatial Fourier spectra of the complex field; (d) scattered intensity versus scattering angle curves obtained from FTLS for varying number of beads as compared to the calculated curve from Mie calculations; and (e) Spectrum of fluctuations in scattering intensity for a system of beads in water for characterizing diffusion coefficient and viscosity. Extraction of scattering parameters through FTLS of rat organ tissue slice: (f) scattering mean-free path $l_{s}$, (g) scattering anisotropy, (h) transport mean-free path $l_{t}$. (i), (j) Phase map and angular scattering results obtained by applying FTLS on rat mammary tumor tissue slice sample over broad range of scattering angles. (a)–(e), (i), and (j) Reproduced with permission from Ref. 15, © 2008, American Physical Society; (f)–(h) reproduced with permission from Ref. 108, © 2009, Optica.

Consider a group of N similar particles distributed in a finite volume as shown in Figs. 14(a) and 14(b).^15^ These particles are assumed to be under random Brownian motion such that their positions are dependent on time and uncorrelated. This dynamic distribution of particles can be summarized as^3^

$$U(r,t)=U_{F}(r,t){Ⓥ}_{r}\overset{N}{\underset{i=1}{\sum }}\delta [r-r_{i}(t)],$$ (39)

where $U_{F}(r,t)$ is the field due to a single particle, $r_{i}(t)$ is the time dependent position of i’th particle, and ${Ⓥ}_{r}$ denotes the spatial convolution. The scattered field is then^3^^,^^15^

$$\overset{˜}{U}(q,t)=\overset{˜}{U_{F}}(q,t)\cdot \overset{˜}{U_{S}}(q,t),$$ (40)

where $\tilde{U_{F}}(q,t)$ is called the form function, which is dependent on the shape of a single particle and thus signifies the overall spatially slowly varying envelope of the total scattered field. The quantity $\tilde{U_{S}}(q,t)=\sum_{i=1}^{N}e^{iq\cdot r_{i}(t)}$ is the structure function, which describes the contribution of the positions of particles on the scattered field.^3^^,^^15^

Time-dependent studies of dynamic systems involve time-lapse image acquisition. The magnitude squared temporal Fourier transform of Eq. (40) yields the power spectral density as^15^

$$P(q,\omega )={\left|\int \overset{˜}{U}(q,t)e^{-i\omega t}dt\right|}^{2}.$$ (41)

Since the fluid dynamics can also be described by a Lorentzian function of the form $1/[1+(\omega /Dq^{2}{)}^{2}]$, where the spectral bandwidth is $Dq^{2}$ and the diffusion constant D is expressed as $k_{B}T/4\pi \eta r_{0}$, with viscosity η and diameter of particle $r_{0}$,^15^ the diffusion coefficient and hence the viscosity can be obtained by fitting the acquired power spectral density data with a Lorentzian curve fit.

An experimental study based on the above discussed principle for a system of multiple 3  μm beads was reported in Ref. 15. There, DPM was used to measure the amplitude and phase information as shown in Figs. 14(a) and 14(b).^15^ Background subtraction was performed by measuring a sample less area and subtracting it from the acquired images. Computing the spatial Fourier transform [Fig. 14(c)] of the measured complex field and averaging along the contours of equal length q, where $q=2\beta_{0}\;\sin(\frac{\theta }{2})$, gives the scattered intensity for every scattered angle θ.^15^ This scattered intensity after normalization is known as the scattering phase function. Figure 14(d) shows the plot of scattered intensity versus the scattering angle for varying number of beads in the field of view.^15^ To compare the accuracy of FTLS measurements, calculations based on Mie theory for 3  μm beads were performed in Ref. 15 and plotted on the scattered intensity plot obtained through measurements. As evident in Fig. 14(d), the oscillations predicted by Mie theory agree well with the FTLS measurements for varying number of beads.^15^ These results showed that FTLS is even sensitive to a single-particle scattering as there is a clear distinction between the background FTLS signal and a single-bead FTLS signal.^15^ Measurements of dynamic system of beads in water are shown in Fig. 14(e),^15^ where the power spectral density P(q,ω) is fitted with the Lorentzian function to extract diffusion coefficient D and viscosity η.

Experimental estimation of scattering parameters from the measured FTLS data was demonstrated in Ref. 108. Considering that the attenuation of the field after passing through a thin tissue slice is due to scattering, the scattering mean free path $l_{s}$ can be extracted from the FTLS measurements by applying Lambert–Beer’s law $I_{0}(L)=I_{0}e^{-L/l_{s}}$, where L is the thickness of the tissue slice, $I_{0}$ is the total incident irradiance, which is a sum of both scattered and unscattered irradiance.^108^ The quantity $I_{0}(L)$ denotes the unscattered irradiance after traversing the tissue and can be calculated by integrating the scattered intensity over the DC or the diffraction spot.^108^ To determine the scattering anisotropy g as defined in Sec. 2.2.2, direct application of Eq. (17) to full thickness of the tissue is not valid. To determine the appropriate g, the complex field can be propagated through $N=l_{s}/L$ layers of tissue slices such that the normalized angular scattering distribution or the phase function can be expressed as^108^

$$p(q)\propto {\left|\iint [U(r){]}^{N}e^{iq\cdot r}d^{2}r\right|}^{2}.$$ (42)

Using Eq. (42), g can be calculated as in Eq. (16).

The range of measured angular spectrum data is limited by the NA of the imaging system (the maximum scattering wavevector q that can be captured and detected by the system), which can result in inaccurate estimates of g. For this reason, Ding et al.^108^ used the Gengenbauer kernel phase function curve-fitting for the measured scattering anisotropy data.^108^ Aided by the measurement of $l_{s}$ and g, the transport mean free path $l^{t}$, which determines the fate of light after traversing a highly scattering media with multiple scattering incidents, can be calculated according to the relation $l^{t}=l_{s}/(1-g)$. Measurements of scattering parameters $l_{s}$, g, and $l_{t}$ for tissue sections from different rat organs are shown in Figs. 14(f)–14(h).^108^ Although the maximum angle is dictated by the optics of the measurement system, FTLS is still able to characterize the scattering behavior of thick tissue slices starting from very low angles as shown in Figs. 14(i) and 14(j), where angular scattering measurements from angles as low as 0.01 deg were made.^15^ This broad range of about three decades of angular scattering data makes FTLS, a unique tool for measuring scattering parameters of the entire organ tissue slice.^15^

## 3 Applications: Cell and Tissue Scattering, Scattering Parameters as Markers of Disease

QPI has found tremendous success in addressing biomedical applications. This success can be attributed to the high phase sensitivity, label-free operation, and noninvasive properties of QPI.^4^^,^^34^^,^^109^ It is known that the dry mass density is linearly proportional to the phase map, and hence through the knowledge of phase obtained through QPI applications, such as monitoring the growth of cells,^50^^,^^110^^–^^117^ understanding brain and neuronal networks,^113^^,^^118^^–^^124^ measurement of cellular dynamics,^50^^,^^115^^,^^116^^,^^119^^,^^123^^,^^125^^–^^129^ organelle detection and characterization,^130^ cancer tissue pathology,^131^^–^^134^ virology,^33^^,^^135^ reproductive science,^136^^–^^141^ etc., have been studied. In addition to the applications discussed with each QPI configuration in Sec. 2, here we present some additional, general applications of QPI in the study of cells and tissues.

### 3.1 Cell Sorting Through FTLS

**Fig. 15:**
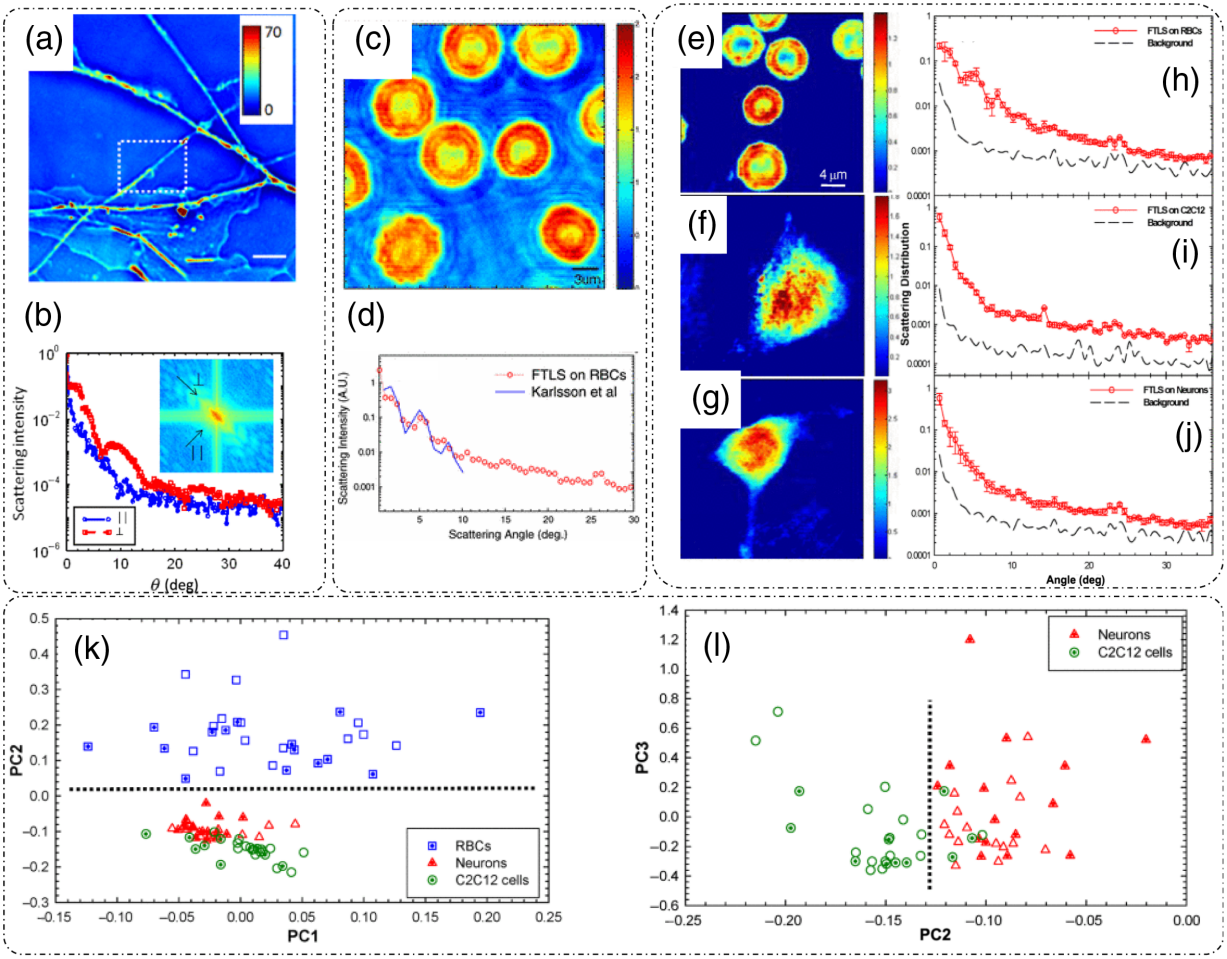
FTLS applications: High dynamic range angular scattering measurements for parts of cells as in (a), (b) neurites to (c), (d) whole cells. Cell sorting: phase maps and scattering phase function for (e), (h) RBCs; (f), (i) C2C12 cells; and (g), (j) neurons. (k), (l) PCA results for cell sorting based on angular scattering measurements through FTLS. (a), (b), (e)–(l) Reproduced with permission from Ref. 142, © 2010, IEEE, with (a) reproduced with permission from Ref. 9, © 2011, Optica. (c), (d) Reproduced with permission from Ref. 15, © 2008, American Physical Society.

Cells can be differentiated from each other using their angular scattering signatures. FTLS provides high sensitivity to measuring scattering signals from very weak scatterers like a neurite [Figs. 15(a) and 15(b)]^9^^,^^142^ and can cover a large range of angles limited only by the optics of the system [Figs. 15(c) and 15(d)].^15^ Ding et al.^142^ proposed a cell sorting technique based on the FTLS measurement of phase functions. Figures 15(e)–15(g) show the measured phase function for three different cell types, RBC, C2C12, and neurons, respectively.^142^ PCA was performed on the angular scattering data for 15 measurements per cell type [Figs. 15(h)–15(j)] and the results are shown in Figs. 15(k) and 15(l).^142^ There is a clear distinction between the scattering signals from three cell types. Ding et al.^142^ reported sensitivity/specificity values of 1/1, 1/0.88, and 0.7/1 for RBC, neurons, and C2C12 cells, respectively. These results, though obtained on a relatively simple classification problem, present the benefit of intrinsic markers as compared to flow-cytometer measurements, which requires fluorescently tagged cells.^142^

### 3.2 Detection of Cancer in Tissues

The standard method of detecting an abnormality in a biopsy is based on inspection of a stained tissue by a trained pathologist. The changes in the tissue preparation can lead to inconsistencies in inference by different pathologists and hence is a potentially subjective procedure. This drawback is associated with every type of sample staining method. To avoid such variability and infer the presence of disease accurately, quantitative markers derived from the intrinsic properties of tissues, like refractive index distribution, are of importance. Since no human intervention is required, any quantitative marker can thus be used in conjunction with modern algorithms for both diagnosis and prognosis of the disease.^132^^,^^143^^–^^146^

The basis for such quantitative markers derived from QPI is that the disease onset and development is accompanied by changes in tissue refractive index, which, in turn, induces changes in light scattering.^145^^–^^147^ QPI measurements provide an optical pathlength difference map, which is highly sensitive, of the order of nanometer.^9^ For a histopathology tissue slice, the thickness is generally known (2 to 4  μm). Thus the optical pathlength map directly corresponds to the refractive index distribution integrated along the beam path in the sample.^146^ Since there is a direct linear relationship between the observed phase difference map and the dry mass density of the sample,^3^ QPI informs not only on the refractive index distribution but also on the density or inhomogeneity of the tissue.^146^^,^^147^

One of the quantitative markers for cancer detection is tissue disorder strength.^147^ In Ref. 147, a study that involved quantitative imaging and analysis of tissue microarray from 400 patients containing both benign and malignant tissue samples was presented. For each core, two adjacent slices were imaged, one stained with H & E and one unstained. From the QPI measurements, the tissue disorder strength was quantified as^147^

$$L_{d}(x,y)=\langle \Delta n(x,y{)}^{2}\rangle_{w}l_{c}.$$ (43)

Here $L_{d}(x,y)$ is the tissue disorder strength, ${\left\langle \Delta n(x,y{)}^{2}\right\rangle }_{w}$ is the windowed spatial variance of refractive index, and $l_{c}$ is the spatial autocorrelation length.

From the measured phase map Δϕ(x,y), $\langle \Delta n(x,y{)}^{2}\rangle_{w}$ can be calculated as^147^

$$\langle \Delta n(x,y{)}^{2}\rangle_{w}=\frac{\langle \Delta \phi (x,y{)}^{2}\rangle_{w}}{{\left(\frac{2\pi }{\lambda }L\right)}^{2}}.$$ (44)

In Eq. (44), $\langle \Delta \phi (x,y{)}^{2}\rangle_{w}$ denotes the windowed spatial variance of the measured phase difference. From Eq. (43), the disorder strength can be expressed as^147^

$$L_{d}(x,y)=\frac{\langle \Delta \phi (x,y{)}^{2}\rangle_{w}}{\langle \phi (x,y)\rangle_{w}^{2}}n_{m}^{2}l_{c},$$ (45)

where $\langle \phi (x,y)\rangle_{w}^{2}=(\frac{2\pi }{\lambda }L{)}^{2}n_{m}^{2}$ is the squared windowed spatial mean of the phase difference, and $n_{m}=\langle n(x,y)\rangle_{w}$ is the windowed average mean of the refractive index in the sample window. As suggested by Eq. (45), another parameter to characterize the tissue disorder strength is spatial autocorrelation length, which can be extracted from the autocorrelation function of measured phase.

**Fig. 16:**
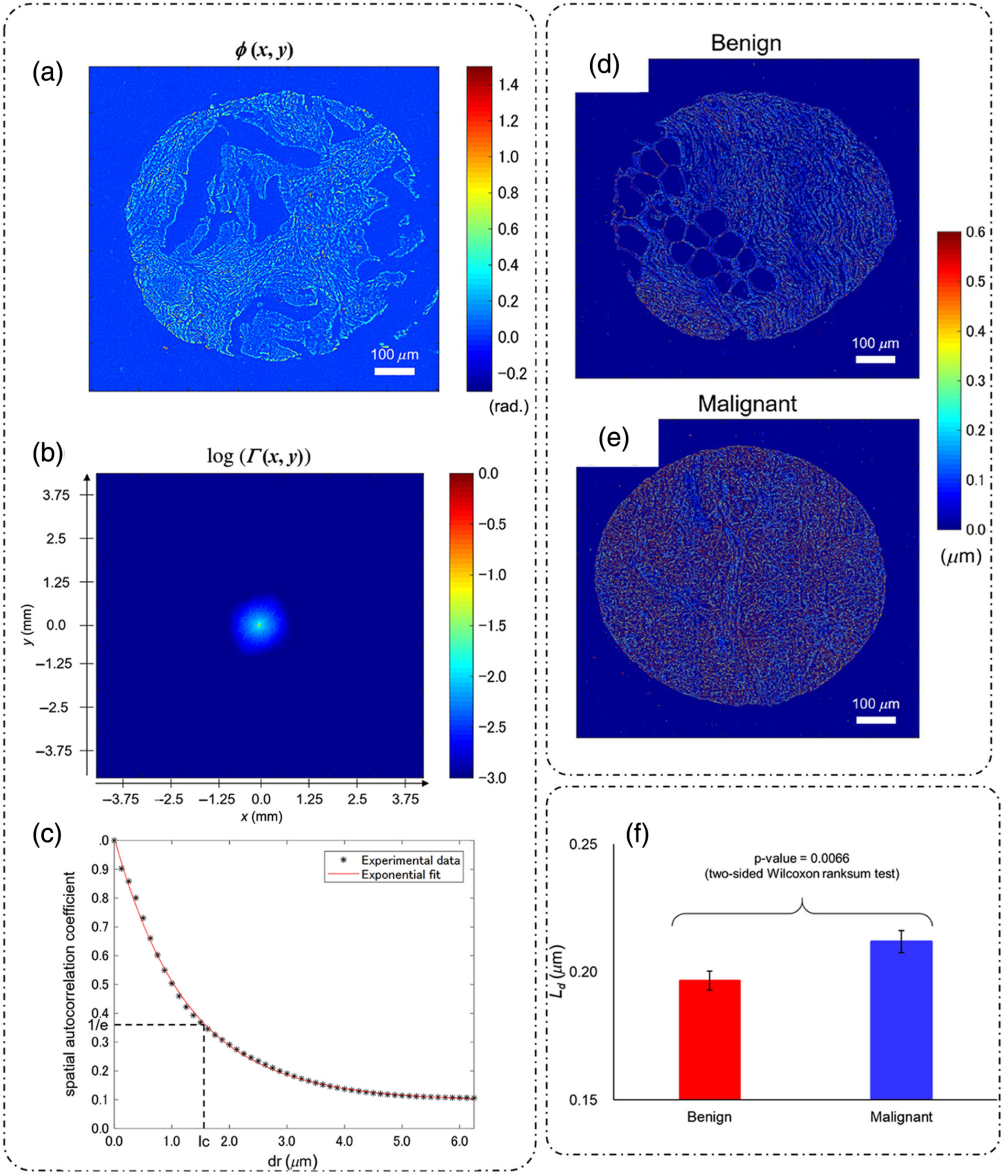
Disorder strength as a cancer marker: (a) tissue biopsy core phase map through SLIM; (b) spatial autocorrelation of phase measurements in (a); (c) 1D plot of (b) for $l_{c}$ extraction; (d) disorder strength map for benign tissue; (e) disorder strength map for malignant tissue; and (f) statistically significant difference in the tissue disorder strength between benign and malignant tissue samples. (a)–(f) Reproduced from Ref. 147 under CC BY license.

Results of the study in Ref. 147 are shown in Fig. 16. Figure 16(a) shows the phase map obtained from SLIM for a tissue biopsy slice of thickness 4  μm.^147^ Figure 16(b) shows the normalized autocorrelation Γ(x,y) of the measured phase difference ϕ(x,y).^147^ Figure 16(c) presents the 1D plot of Fig. 16(b), where spatial correlation length is defined as the distance (x axis), in which the autocorrelation falls to 1/e of its maximum value.^147^ Based on the expressions mentioned above, the disorder strength map was extracted in Ref. 147 from the SLIM measurements and is shown in Fig. 16(d) for benign and Fig. 16(e) for malignant tissues.^147^ It is evident that the tissue disorder strength is higher in the malignant tissue slice. A statistical justification of this observation is presented in Fig. 16(f),^147^ where the bar graph shows the measured tissue disorder strengths for 20 benign and 20 malignant tissues. The difference between the two groups was statistically significant with a p-value of 0.0066 obtained by applying two-sided Wilcoxon rank-sum test to the data.^147^ The implication of Fig. 16(f) is that the tissue disorder strength is a potentially significant intrinsic marker for malignancy in tissues.^147^

Comparing Eq. (45) (for disorder strength) and Eq. (12) (for scattering mean free path), the scattering mean free path and disorder strength are inversely proportional to each other.^147^ This implies that the higher the disorder strength is, the lower the scattering mean free path is, and, hence, the stronger the scattering will be. The stronger scattering also indicates that there is a high degree of inhomogeneity in the refractive index of malignant tissues.^147^

**Fig. 17:**
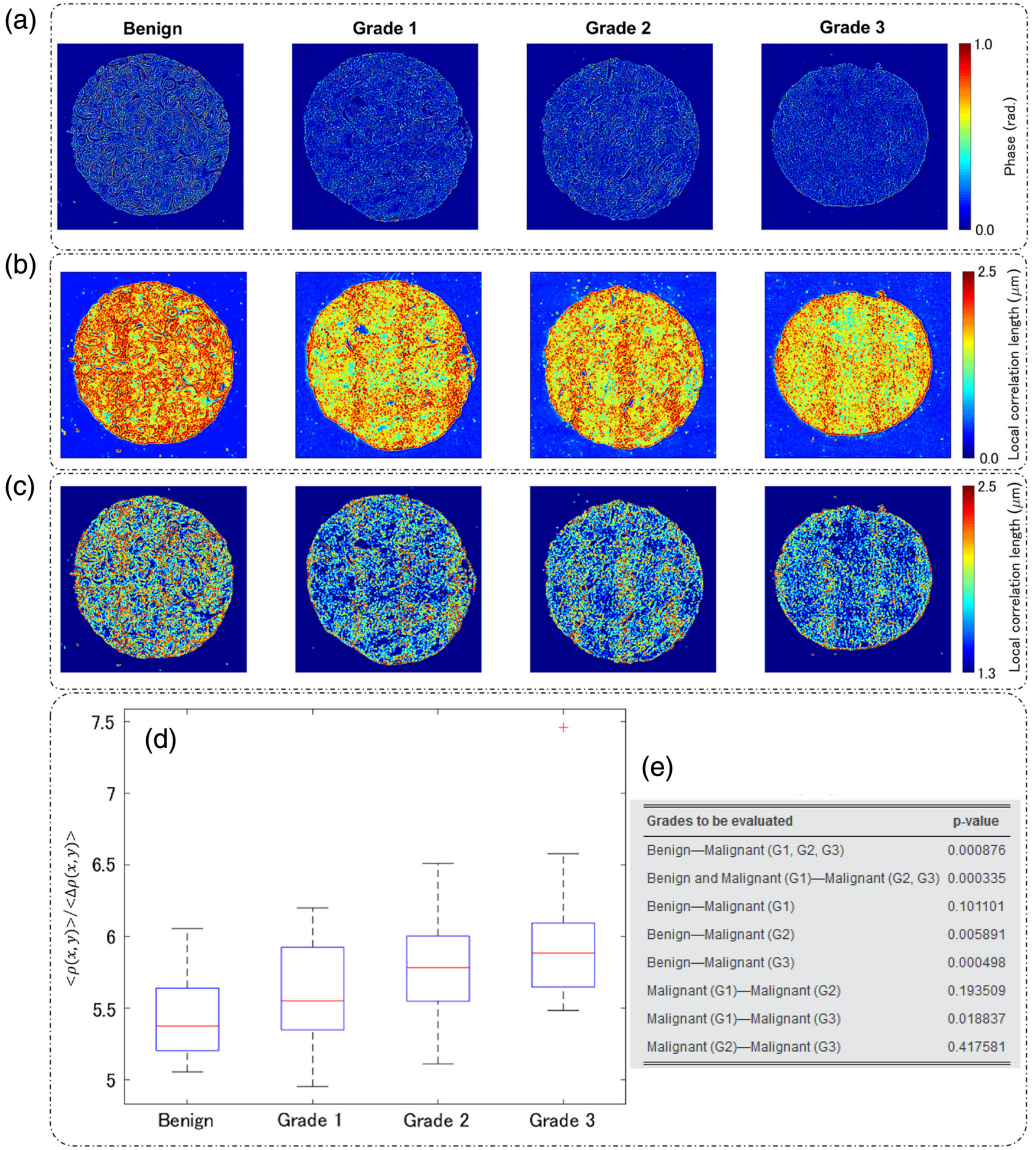
Tissue spatial correlation length as cancer marker: (a) phase measurements through SLIM for benign and three grades of malignant tissue cores; (b) local autocorrelation length maps; (c) thresholded local autocorrelation length maps; (d), (e) differentiation of benign versus malignant and differentiation of grades of malignancy based on local autocorrelation lengths with their respective p values as an indicator of success or failure per case. (a)–(e) Reproduced from Ref. 146 under CC BY license.

Another intrinsic cancer marker is the local spatial autocorrelation length.^146^ Figure 17 shows that the local spatial autocorrelation length [full and thresholded in Figs. 17(b) and 17(c), respectively] derived from phase measurements [Fig. 17(a)] can be employed to detect and differentiate between benign [first column, Figs. 17(a)–17(c)] and different grades of malignant tissue [rest three columns, Figs. 17(a)–17(c)].^146^ The boxplot [Fig. 17(d)] and the statistical significance test results [Fig. 17(e)] show that except for three cases [benign–malignant (G1), malignant (G1)–malignant (G2), malignant (G2)–malignant (G3)], all the rest combinations are significantly different.^146^

### 3.3 Assessment of Injuries

Like the gold standard detection methods for cancer, the evaluation of acute kidney injury also requires examination of H & E-stained tissue sections by a trained physician. Current imaging modalities are limited by either spatial resolution (MRI, PET) or require extensive staining or sample preparation (μCT, fluorescence imaging, etc).^148^ Understanding the scattering properties of injured kidney tissue and extracting intrinsic markers to study disease progression through high spatial resolution QPI techniques will eliminate the shortcomings of the previously mentioned modalities. In Ref. 148, DPM was employed as a QPI instrument to extract phase and hence scattering properties associated with thin (5  μm) injured kidney tissue slices.

### 3.4 Assessment of Type and Grade of Breast Cancer

Studies indicate that there is an increased and observable change in the extracellular matrix or stroma of the diseased tissues as the tumor progresses.^149^^,^^150^ The arrangement of collagen fibers in the extracellular matrix is an indication of progression of stage of the tumor.^151^ Since collagen fibers have strong second-order nonlinear susceptibility $\chi^{\left(2\right)}$, second harmonic generation microscopy (SHGM) has been the most popular tool to assess the fibers in relation to tumor growth. However, SHGM cannot provide a holistic view of biopsy due to the presence of centrosymmetric structures, such as epithelial tissues.

Thus a modality that can detect and quantify both centrosymmetric and noncentrosymmetric structures is much more desirable. It was shown in Ref. 151 that SLIM can generate the orientation map of collagen fibers with accuracy comparable to SHGM measurements. In addition, SLIM can also provide cellular information for centrosymmetric structures that SHGM cannot. In addition to the above advantage, SLIM has other advantages over SHGM in terms of hardware requirements and ease of measurements. While SHGM requires expensive and bulky femtosecond laser sources that are inherently high peak power sources, SLIM operates with white light, broadband sources like halogen lamps or LEDs, which are amenable to observation for larger durations since the amount of illumination power delivered is much less than the damage threshold of the sample under study. Also SLIM is a widefield measurement method as compared to point scanning SHGM modality, which in turn increases the throughput.^151^

**Fig. 18:**
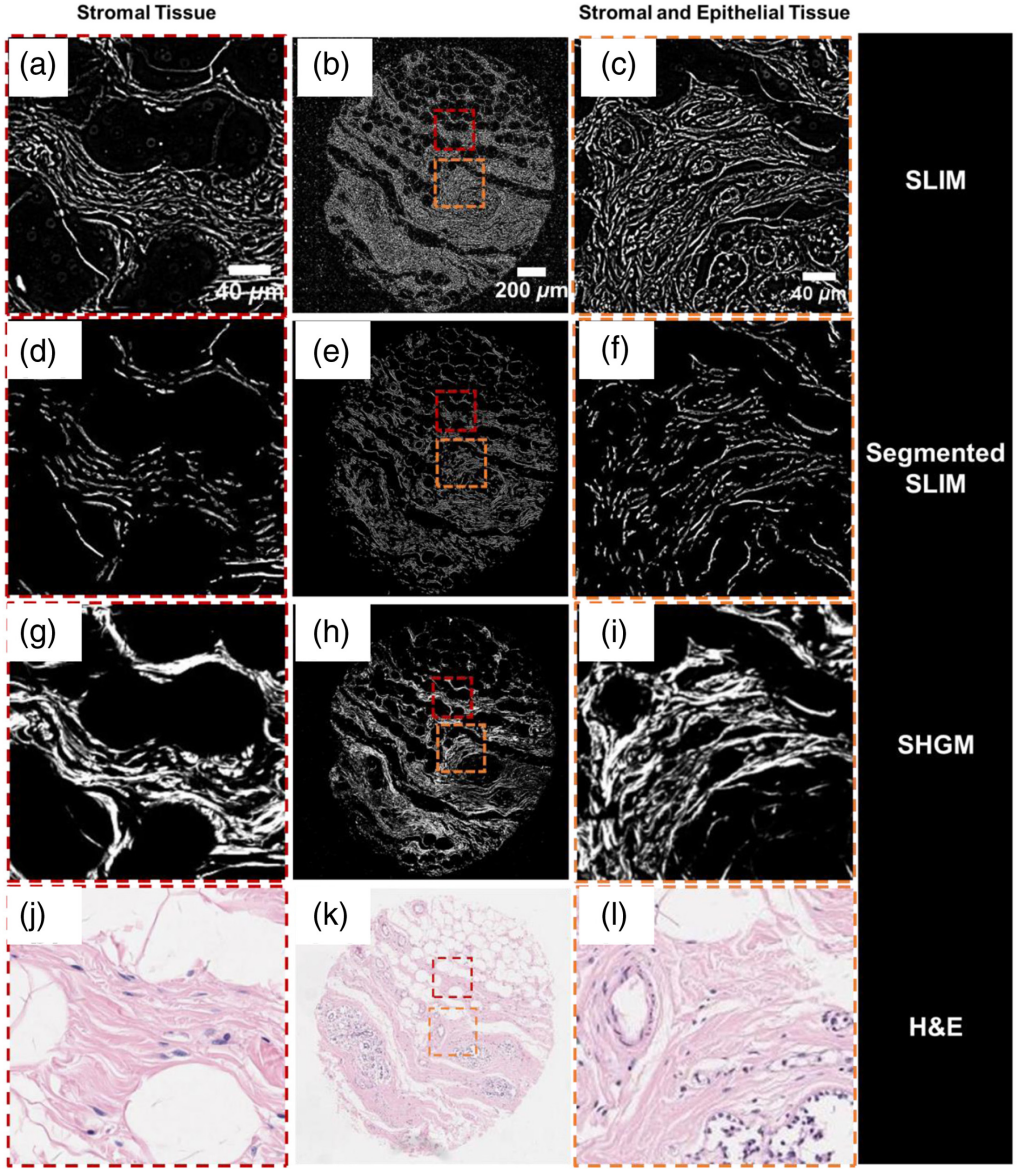
SLIM application for grading cancer based on collagen information. SLIM images (first row), segmented SLIM images (second row), SHGM images (third row) and H&E images (fourth row) of (a), (d), (g), (j) stromal tissue region; (b), (e), (h), (k) full core; and (c), (f), (i), (l) stromal and epithelial tissue regions. (a)–(l) Reproduced with permission from Ref. 151, © 2017, SPIE.

To prove that SLIM can provide information about collagen fibers similar to the SHGM, an SLIM, H & E, and SHGM investigation of a 24-core TMA with 8 benign and 16 malignant cores of varying grades was carried out in Ref. 151. To quantify the fiber orientations from SLIM images, isotropic measurements due to centrosymmetric structures, such as epithelial tissues, were segmented out using a set of filters and image gradient-based segmentation algorithms as discussed in Ref. 151. Figures 18(a)–18(c) show the SLIM image (top row) for two different regions of the same core [Fig. 18(b)] highlighting mostly anisotropic structures in the first column [Fig. 18(a)] and mixed structures in the third column [Fig. 18(c)].^151^ Figures 18(d)–18(f)^151^ show the segmented SLIM to recover fibers, which is comparable to SHGM images in Figs. 18(g)–18(i).^151^ The presence of isotropic structures in SLIM [Figs. 18(a)–18(c)], such as cells and epithelial tissue, is confirmed through H & E images in Figs. 18(j)–18(l).^151^

### 3.5 Cellular and Organelle Investigations, Integration with Machine Learning

QPI has been used extensively for characterizing cellular and organelle perturbations. Few examples include characterization of the effect of bacteria on human primary T-cells using time lapse DHM,^152^ where the T-cells displayed changes in morphology (area and circularity) and mean phase contrast in response to the bacterial stress, indicating the utility of QPI in assessing host-pathogen interplay in a label-free manner. QPI has also been employed to study intracellular components like lipid droplets in live microalgal culture cells.^153^ Giugliano et al.^154^ recently demonstrated the ability of QPI in detecting and characterizing lysosomal compartments in mouse embryonic fibroblasts between different populations. Such a characterization of lysosomal compartments can provide meaningful information about lysosomal storage diseases. Recently, the QPI techniques have gained enhanced cellular and subcellular specificity with the rapid advent of deep learning.^8^ These computationally enhanced QPI techniques are making significant advances from basic science-in detection and characterization of intracellular organelles in a label-free manner,^155^ study of embryos for health grading,^140^ to pathology-in detection of hematologic disorders,^156^ and finally toward clinical applications-in detection and classification of cancer cells.^157^

## 4 Conclusions

In this paper, we reviewed the state-of-the-art in QPI techniques for biomedical applications. We started with explanation and mathematical description of phase measurements for thin tissue slices in both forward and backscattering cases and reviewed 2D scattering phase theory. A detailed review of 2D phase imaging techniques based on holographic and interferometric principles of measurement was provided. Fourier transform light scattering was reviewed. Toward the end, we discussed a few applications of the scattering measurement techniques for characterizing cells and tissues.

## Data Availability

No new data were generated in this study. No codes were used for this study.
